# The hidden challenges of return migration: Linguistic and academic re-adaptation of Syrian students to the Arabic Education System

**DOI:** 10.1371/journal.pone.0361042

**Published:** 2026-10-16

**Authors:** Sevda Dolapcioglu, Yunus Emre Akbana, İlayda Uzun, Gülten Koşar

**Affiliations:** 1 Hatay Mustafa Kemal University, Department of Education Science, Hatay, Türkiye; 2 Kahramanmaraş Sütçü İmam University, Department of English Language Teaching, Kahramanmaraş, Türkiye; 3 Republic of Türkiye, Ministry of National Education, Hatay, Türkiye; 4 Hatay Mustafa Kemal University, Department of English Language Teaching, Hatay, Türkiye; Mersin University: Mersin Universitesi, TÜRKIYE

## Abstract

While extensive research has examined refugee students’ educational adaptation in host countries, the educational reintegration phase following voluntary return remains underexplored. This qualitative study investigates the linguistic and academic adaptation experiences of Syrian students transitioning from Turkish-medium education in Türkiye to Arabic-medium schooling in Syria after voluntary return. The study addresses an overarching question: how do returnee students experience the linguistic, academic, social, and psychosocial dimensions of this reverse educational transition? Using an exploratory qualitative design, data were collected through a seven-item open-ended written questionnaire from 15 middle-school students who had received several years of formal schooling in Türkiye. Qualitative content analysis revealed that return does not constitute a simple linguistic recovery, but rather a reverse academic transition characterized by profound difficulties with academic Arabic, conceptual transfer, written production, and in-class assessment practices. The findings indicate that students’ challenges were shaped not only by uneven Arabic literacy development but also by curriculum misalignment, pedagogical practices that participants perceived as more teacher-directed, limited peer interaction, and assessment practices that they perceived as unclear. Furthermore, psychosocial pressures, including re-migration-related distress, concentration difficulties, and increased family responsibilities, complicated their academic adaptation. Ultimately, these struggles reflect systemic institutional and contextual mismatches rather than individual deficits. By conceptualizing return as a distinct educational phase, this study contributes to the broader discourses on refugee education and bilingualism. The implications underscore the critical need for academic Arabic bridging programs, background-sensitive assessment frameworks, psychosocially responsive pedagogies, and school-based psychosocial support to ensure equitable reintegration into Arabic-medium schooling.

## Introduction

The Syrian conflict that began in 2011 has reshaped schooling trajectories for millions of children across the region [1]. Türkiye has been a major host country, and large numbers of Syrian children have spent formative years within Turkish society and Turkish-medium education [2,3]. While much research has focused on educational access and adaptation during displacement, an increasingly visible development is voluntary return, particularly to parts of northern Syria, and the resulting return of students from Turkish-medium schooling back into Arabic-medium schooling [4]. This transition is more than a change of country or curriculum. For many children, it involves rebuilding academic participation through a language that may be culturally familiar yet educationally underdeveloped.

A key reason this return context matters is that language is not only a subject in school; it is the medium through which learning, assessment, and classroom belonging are negotiated [5]. Children who have been educated in Turkish for years often develop Turkish as their dominant language for academic thinking and school routines, while Arabic may be maintained unevenly—sometimes limited to home interaction, sometimes reduced further due to schooling conditions and peer ecologies [6,7]. Research on immigrant and refugee learners repeatedly shows that academic language proficiency develops more slowly than everyday conversational fluency and is shaped by both instruction and interactional opportunities [5,8]. This is crucial for returnees entering Arabic-medium schools since the demands of schooling are heavily literacy- and assessment-driven; difficulties may therefore surface in academic vocabulary, written expression, subject learning, and test performance, even when children can manage basic spoken interaction.

At the same time, return transitions unfold under post-conflict and post-displacement pressures that can affect learning in school. Studies of war-affected populations highlight that mental health and adjustment are shaped not only by traumatic exposure but also by daily stressors and psychosocial conditions in post-conflict settings [9]. In educational terms, refugee and return contexts often require learners to manage stress, uncertainty, and identity negotiation alongside academic expectations [10,11]. International policy discussions on crisis education and post-conflict recovery also emphasize the role of supportive school climates and trauma-informed practices in protecting learning and wellbeing [12]. For children returning to Arabic-medium schooling after years of Turkish-medium education, the classroom can therefore be both a site of language development and a site where vulnerability is intensified or reduced, depending on support.

A substantial body of research on Syrian refugee children addresses host-country language acquisition and Arabic maintenance/attrition during the host-country stay period, identifying consistent predictors such as age-related variables, input conditions, peer networks, and educational opportunity structures [6,13-16]. However, much of this work focuses on children who had acquired at least some Arabic before migration and then experienced Arabic under reduced input abroad [7,13]. Far less is known about children born and raised in Türkiye who develop Turkish as their dominant language and later return to Syria, encountering Arabic-medium schooling as an academic language for the first time [7]. This gap is crucial because it shapes how schools interpret learners: without a research-based account of the returnee profile, schools may treat difficulties as low ability rather than as predictable outcomes of bilingual development under displacement and return.

This study responds to that gap by focusing on Syrian returnee students moving from Turkish-medium schooling in Türkiye to Arabic-medium schooling in Syria after voluntary return. More specifically, this study aims to examine the linguistic and academic adaptation experiences of Syrian students during their transition from Turkish-medium schooling in Türkiye to Arabic-medium schooling in Syria after voluntary return. The study takes a school-based view of language development: it examines how returnee learners experience Arabic as the medium of instruction, how difficulties appear in classroom participation and assessment, and what contextual conditions (family language environments, peer networks, instructional support) shape adaptation. By doing so, the study extends refugee language research beyond host-country integration to capture a neglected stage of the migration trajectory; namely the return phase, where bilingual profiles shaped in the host-country stay period meet the immediate demands of Arabic-medium schooling [4,7]. Accordingly, the study was guided by the following overarching research question: How do Syrian middle-school students experience the linguistic and academic re-adaptation process during their transition from Turkish-medium education in Türkiye to Arabic-medium schooling in Syria after voluntary return?

This overarching question was addressed through four sub-questions:

(1) What academic-language-related challenges do returnee students experience in Arabic-medium instruction?
(2) How do returnee students experience social integration and academic confidence in their new school environment?
(3) What challenges do returnee students encounter in re-adapting to the curricular, pedagogical, and assessment practices they experience in their post-return schools?
(4) How do psychosocial factors shape returnee students’ post-return academic experiences?

## Literature review

Language in school is inseparable from learning and assessment. In bilingual education and multilingual schooling research, academic language is not treated as a simple extension of everyday fluency; it is a distinct register tied to literacy, subject concepts, and institutional expectations [5]. This distinction is most systematically articulated in Cummins’ [17] differentiation between conversational fluency and academic language proficiency: while everyday communicative skills may develop relatively quickly through social interaction, the cognitively demanding and context-reduced language of schooling requires considerably longer and depends heavily on sustained literacy engagement and instructional support. Cummins [17] further argues that academic skills developed in one language can support learning in another, but only when learners are given the linguistic tools and opportunities to access that prior knowledge. Such a distinction is central to return contexts because returnee students must demonstrate knowledge through Arabic immediately, often without a long transition period. Research on academic achievement in a second language suggests that developing academic proficiency typically requires sustained time, rich instructional support, and stable schooling conditions [8]. When schooling experiences are disrupted or when the language of instruction changes abruptly, learners may be positioned as struggling even when their underlying cognitive ability is intact [18]. These insights support a key premise: returnee challenges can be best understood through the classroom, where language, literacy, assessment, and belonging converge.

### Heritage language pathways

For Syrian children raised in Türkiye, Arabic often functions as a home language with reduced institutional reinforcement, while Turkish becomes dominant through schooling and peer interaction [2,19]. Heritage language theory provides a useful perspective for this ecology. Heritage languages may diverge from monolingual baselines under reduced input and limited literacy opportunities, and outcomes vary substantially across learners [20,21]. Importantly, bilingual systems are dynamic: later-learned dominant languages can reshape earlier linguistic representations, and vulnerability can differ across domains [22]. The focus on Syrian refugee children’s bilingual development makes this heterogeneity concrete. Studies show that Arabic outcomes depend on age-related variables, language environment factors, and cognitive capacity [6,15]. Longitudinal evidence highlights that Arabic can weaken over time when schooling and broader environments shift to the host language, and that stronger Arabic outcomes are associated with richer Arabic use, educational opportunity, and family resources [13]. From the standpoint of return migration, the implication is direct: children will not enter Syria with the same Arabic “starting point,” and school-based difficulties are likely to reflect earlier exposure patterns rather than simply effort or motivation [7].

### After return: Reactivation and reversal

Returnee research in other linguistic communities demonstrates that weakened childhood languages can regain strength when the sociolinguistic ecology shifts and the heritage language becomes the majority language again [21,23,24]. Evidence from returnee contexts (e.g., Turkish-German, Portuguese-German) suggests that children and adolescents may show rapid gains in vocabulary and core morphosyntax after return, sometimes approaching monolingual-like performance within a few years [25–27]. However, the literature also cautions that recovery can be domain-specific: more complex or discourse-sensitive targets may remain vulnerable even with sustained immersion, and outcomes depend on prior exposure and continued language use in social networks [21,25]. For Syrian returnees from Türkiye, this means that Arabic-medium schooling can indeed provide dense input, but it does not guarantee smooth academic adjustment. If a child’s Arabic before return was mostly oral and limited in academic literacy, the classroom may quickly expose gaps in written expression and curriculum-specific vocabulary. In other words, “re-immersion” can support growth while simultaneously producing academic risk when school expectations outpace linguistic resources [5,8,21].

### When post-return Arabic functions as a second language

For children born and raised in Türkiye with minimal Arabic exposure, post-return Arabic development often resembles Second Language Acquisition (SLA), particularly in the academic register required for schooling. SLA syntheses emphasize that progress depends on meaningful input and on interactional conditions that make input usable; especially feedback, opportunities to negotiate meaning, and structured instructional support [28–30]. Sociocultural and interactionist perspectives further highlight guided participation and scaffolding; for example, learners develop through participation in communities of practice where more capable peers/teachers support performance beyond current independent ability [30,31]. In Arabic-medium schooling, this points to a practical mechanism. That is, returnee trajectories are shaped not just by exposure to Arabic, but by whether classrooms provide scaffolded access to academic talk, reading and writing tasks, and assessment formats. This point connects closely to what refugee bilingual development studies show about predictors of outcomes. Across Syrian refugee samples, bilingual profiles are linked to age variables, learning conditions, and cognitive resources [6,15]. Such findings align with the expectation that returnees will differ in how quickly they can move from basic communicative ability to academic language performance. For many, the major challenge is not speaking Arabic in everyday contexts; it is using Arabic to learn and demonstrate subject knowledge through literacy-based tasks.

### Refugee- and return-specific constraints

A recurring issue in language learning research is that refugee learners are often grouped with immigrants despite distinct constraints affecting learning opportunities [19]. Specifically in the Turkish context, studies highlight how discrimination and structural barriers shape educational access and language learning conditions [3,32,33]. Such structural instability often leads to disrupted schooling, which can weaken literacy foundations and academic routines, making later transitions more difficult [2,34]. Moreover, acculturation-oriented research links these integration experiences to motivation and opportunities for language learning, suggesting that bilingual profiles formed in the host-country stay period can persist and carry into the return phase [19,35]. Beyond schooling continuity, psychosocial conditions are equally critical. Specifically, trauma-focused frameworks emphasize that war exposure and daily stressors jointly shape wellbeing and functioning in conflict settings [9]. In this context, refugee education research highlights that learning is intertwined with protection, belonging, and institutional care [10]. Furthermore, from the perspective of immigrant youth development, school adaptation extends beyond academics to involve social relationships, identity work, and the rebuilding of confidence [11]. Consequently, policy-facing work on crisis education emphasizes trauma-informed practices and inclusive approaches that support resilience [12,36]. Together, these sources support a key claim for returnees. Arabic-medium schooling difficulties can be intensified by stress and uncertainty, but supportive teacher/peer relations and school practices can also act as protective factors. Relying on the evidence from the abovementioned pertinent literature, three consistent expectations emerge for Syrian returnee children moving from Turkish-medium schooling to Arabic-medium schooling after voluntary return. First, returnees bring heterogeneous starting points in Arabic shaped by earlier input conditions, schooling opportunities, and cognitive resources [6,7,13,15]. Second, re-immersion can support growth but does not ensure a complete academic recovery; domain-specific vulnerabilities, especially in academic language and literacy, are likely when school demands outpace linguistic resources [8,21,25]. Third, school context is decisive. In other words, scaffolding, interactional access, assessment practices, and psychosocial support shape whether Arabic-medium schooling becomes a pathway to development or a site of cumulative academic risk [10,12,14,31]. Building on this foundation, this study focuses directly on the return phase that remains under-examined in terms of the linguistic and academic adaptational experiences of Syrian students who transition from Türkiye’s education system to Syria’s Arabic-medium schooling after voluntary return.

## Methods

### Research design

An exploratory qualitative approach was adopted. Qualitative inquiry seeks to understand how individuals experience a particular phenomenon, how they perceive it, and the meanings they attribute to it [37]. Accordingly, this study aimed to develop an in-depth understanding of challenges students encounter during the transition into Arabic-medium schooling, grounded in their subjective experiences. This approach enables the researchers to examine participants’ experiences directly from their own perspectives and to interpret the resulting data within their social, cultural, and pedagogical context.

The use of an exploratory qualitative design was considered suitable because the study sought to explore students’ lived experiences, perceptions, challenges, and meaning-making processes during this educational and linguistic transition. Rather than aiming for statistical generalization, the study aimed to generate an in-depth and context-sensitive understanding of the academic, linguistic, and psychosocial challenges encountered by a specific group of students.

Although qualitative inquiry often employs interviews, the geographical separation of participants and the post-return context made face-to-face interviews impractical. Therefore, open-ended written responses were used as an alternative means of accessing participants’ experiences. The use of qualitative content analysis was also appropriate because it enabled the researchers to systematically organize students’ written narratives into initial codes, categories, and overarching themes while preserving the interpretive orientation of the study [38]. In this sense, content analysis served as the analytic procedure through which the meanings embedded in participants’ experiences were identified and interpreted.

### Participants

In this study, criterion sampling, a purposive sampling technique, was employed. This method involves selecting participants who meet predetermined characteristics relevant to the research purpose [39]. The participant group consisted of 15 Syrian students who, after receiving education in Türkiye, voluntarily returned to Syria and attempted to integrate into Arabic-medium schooling during the time of data collection. The inclusion criteria were as follows:

1. Being a Syrian student who had previously received Turkish-medium education in Türkiye,
2. Having returned to Syria in 2024 or 2025,
3. Being enrolled in or attempting to adapt to Arabic-medium schooling after return, and
4. Being at the lower-secondary/middle-school level.

The participants were between 11 and 14 years old. Although this age range may limit the degree of abstract reflection expected in adult qualitative studies, middle-school students are capable of expressing meaningful reflections on their school experiences, academic challenges, peer relations, teacher support, and language-related difficulties when questions are age-appropriate and clearly formulated. Therefore, the questions were designed in a simple, concrete, and experience-based manner to elicit accessible but meaningful qualitative data from young participants.

To protect confidentiality, participants were coded as S1 through S15. Prior to return, all students were enrolled at the lower-secondary (middle school) level in a public school in Türkiye. The participants were 11- to 14-year-old middle-school students selected from individuals who migrated to Türkiye between 2015 and 2019 and then returned to Syria in 2024 and 2025. Details of the study group are presented in Table 1. Within this criterion-defined pool, the final sample reflected the eligible students who were accessible and voluntarily participated during the predefined recruitment period; recruitment was not terminated on the basis of prospective saturation.

**Table 1:** Participant characteristics.

Participant	Gender	Grade Level	Year of Migration to Türkiye	Year of Return to Syria
**S1**	F	6	2017	2024
**S2**	F	8	2016	2025
**S3**	F	6	2015	2025
**S4**	M	8	2016	2024
**S5**	F	7	2017	2024
**S6**	F	7	2018	2025
**S7**	M	7	2016	2024
**S8**	M	6	2019	2025
**S9**	F	8	2018	2025
**S10**	M	6	2019	2025
**S11**	M	5	2019	2025
**S12**	M	8	2015	2024
**S13**	F	7	2017	2024
**S14**	F	6	2017	2024
**S15**	M	7	2017	2025

### Data collection

An open-ended written questionnaire administered via Google Forms was employed as the primary data collection instrument. The use of an online written form was necessary because the participants had returned to Syria and it was not feasible for the researchers to conduct face-to-face interviews. Although this method limited opportunities for follow-up questioning and probing, it enabled the researchers to reach participants who were geographically inaccessible and allowed students to respond in a familiar and flexible format. The questions were developed through a review of prior studies addressing comparable themes and were subsequently refined based on expert feedback to establish content validity. To evaluate the clarity and functionality of the protocol and to enhance the credibility of the data collection process, a pilot questionnaire was administered to two students from the target group who were not included in the data analysis section of this study. In addition to the expert review conducted for content validity, this pilot administration served to establish the face validity of the protocol: the two pilot students evaluated whether the questions were clear, comprehensible, and meaningful from the perspective of the target age group, and their feedback informed minor wording revisions. Completing the questionnaire took participants approximately 15–25 minutes. No incentives or compensation of any kind were provided to participants. The final protocol consisted of seven open-ended questions aligned with the research questions. The questions were as follows:

1. After receiving Turkish-medium education in Türkiye, what were the main academic difficulties you experienced when you transitioned to Arabic-medium schooling in Syria?
2. Were there topics you found difficult to understand in Arabic-medium lessons? In which subjects did you experience these difficulties most?
3. After Turkish-medium education, did you experience difficulty in learning and using Arabic academic terminology? Could you provide examples?
4. Did you notice a difference between your written and spoken Arabic skills? Which skill area was more challenging for you, and why?
5. After moving to the Arabic-medium education system, did your academic achievement change? What do you think explains this change?
6. During your adaptation to Arabic-medium schooling, how did your teachers and peers respond to you? How did these attitudes affect your motivation?
7. After transitioning to Arabic-medium schooling, did you notice any change in your academic self-confidence? How would you explain these changes?

The questions were presented in both Turkish and Arabic, and participants were allowed to respond in either language. The Google Forms link was distributed via WhatsApp with the assistance of students’ parents or legal guardians. All responses were recorded and stored by the research team.

For analysis and reporting, participants’ responses were translated into English by researchers with academic expertise in language studies. To ensure translation accuracy, a back-translation procedure was employed. Another multilingual researcher fluent in Turkish, Arabic, and English reviewed the translated excerpts. Any discrepancies were discussed until consensus was reached. Quotations presented in the findings were selected based on analytic relevance and were checked to preserve participants’ intended meanings rather than literal word-for-word equivalence.

### Ethical considerations

Ethical approval for this study was granted by the Social and Human Sciences Scientific Research and Publication Ethics Committee of Hatay Mustafa Kemal University on January 2, 2026 (Decision No: 31, Document No: 902–01-FR 006). Following this ethical clearance, participant recruitment and data collection were conducted from January 12, 2026, to January 30, 2026. Prior to data collection, written informed consent was obtained from the parents or legal guardians of all participating minors. Furthermore, verbal assent was secured from the children themselves prior to completing the questionnaire; this assent was documented by the researcher to verify and ensure the voluntary nature of their participation. Participants were informed of their right to withdraw from the study at any stage without penalty and were briefed on the measures taken to maintain confidentiality. To protect participants’ identities, all data were anonymized, and pseudonyms (S1–S15) were used throughout the manuscript.

### Trustworthiness and credibility

Several strategies were employed to enhance the trustworthiness and credibility of the qualitative analysis in line with Elo et al.’s [38] model for qualitative content analysis. First, an audit trail was maintained to document coding decisions, category development, and theme refinement. Second, the coding process was conducted systematically using MAXQDA 2020. Third, a second coder independently reviewed 40% of the responses to assess code consistency and analytic coherence. Intercoder reliability was calculated using the Miles and Huberman formula, yielding an agreement coefficient of 86%, which indicated satisfactory coding consistency [40]. Disagreements were resolved through discussion and consensus rather than through statistical agreement, in line with the interpretive nature of qualitative inquiry.

Fourth, preliminary findings were shared with four participants for member checking. This process enabled participants to confirm whether the interpretations accurately reflected their experiences and to clarify meanings where necessary. Finally, the researchers engaged in repeated discussions during the development of codes, categories, and themes. These procedures contributed to the credibility, dependability, confirmability, and transparency of the analysis.

### Data analysis

The qualitative data were analysed using qualitative content analysis. Coding commenced after the data-collection period had ended and the complete set of 15 responses was available; thus, coding did not occur concurrently with participant recruitment. All 15 responses were subsequently analysed in an analytic sequence, and this sequence played no role in determining recruitment or sample size. Qualitative content analysis enables researchers to organise data by identifying recurrent meanings and grouping them into initial codes, categories, and overarching themes [38]. In this study, the analysis followed several stages. First, all responses were read repeatedly to gain familiarity with the data. Second, meaningful units related to students’ academic, linguistic, social, and emotional experiences were identified and assigned initial codes. Third, conceptually similar codes were grouped into categories. Fourth, the categories were organized into four overarching themes. Finally, the themes were interpreted in relation to the research questions and the broader phenomenon of transition from Turkish-medium to Arabic-medium schooling.

MAXQDA 2020 was used to support data organization, coding, and retrieval. Code-frequency outputs were used as an organizational aid; however, frequency was not treated as evidence of thematic importance. Instead, analytic attention was given to the meaning, depth, and relevance of participants’ responses. This approach allowed the researchers to present the findings in a structured manner while preserving the interpretive depth required in qualitative research.

## Findings

The findings emerged under four main themes: difficulties in academic language adaptation, social integration and academic confidence, re-adaptation to the education system, and psychosocial and contextual pressures. Fig 1 illustrates the thematic mapping developed through content analysis:

**Fig 1:**
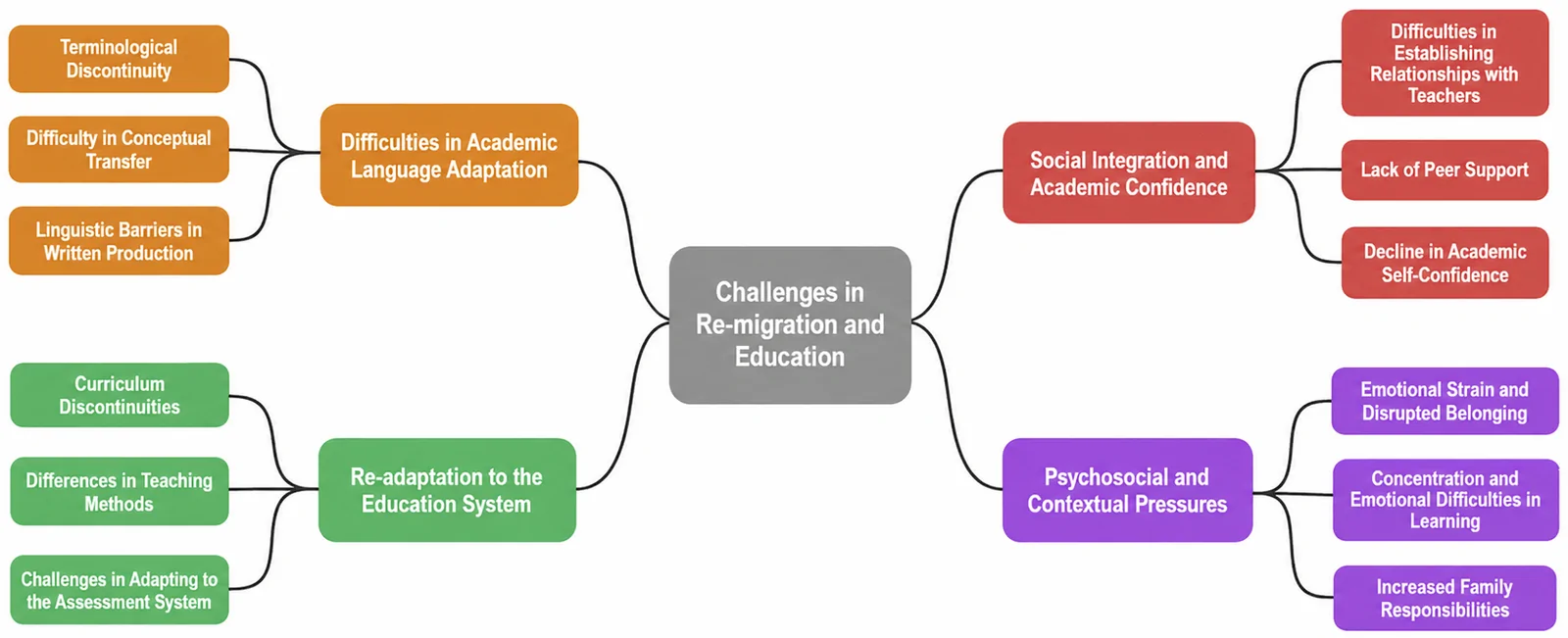
The overall themes and categories.

### Theme 1: Difficulties in academic language adaptation

Students returning to Syria reported that they experienced difficulties, particularly with academic content, due to the change in the language of instruction from Turkish to Arabic. This situation is a common experience not only for students who speak everyday Arabic, but also for many students who struggle to structure academic language. In this theme, how students’ language skills were challenged in the process of adapting to the Arabic educational environment was analyzed within the framework of three categories: terminological discontinuity, difficulty in conceptual transfer, and linguistic barriers in written production. Table 2 shows the categories, codes, and sample data extracts below:

**Table 2:** Difficulties in academic language adaptation.

Category	Codes	Sample Data Extract
**Terminological Discontinuity**	Unfamiliarity with technical terminology; alienation from subject-specific vocabulary; relearning concepts; misinterpretation of terms.	“I learned science in Turkish, but when it is taught in Arabic, new words come up and I don’t understand what they mean.” (S6)
**Difficulty in Conceptual Transfer**	Mental translation; dependence on Turkish-based schemas; breakdown of conceptual maps; difficulty in reconstructing meaning.	“I try to understand the lesson by translating it into Turkish in my head, and this slows me down.” (S5)
**Linguistic Barriers in Written Production**	Difficulty in formulating sentences; limited grammatical control; fear of mistakes; reduced written expression	“I have ideas, but I cannot form sentences in Arabic when writing.” (S2)

#### Terminological discontinuity.

The vast majority of students noted that they were unfamiliar with the Arabic equivalents of the terms used in technical courses and therefore had to relearn the concepts. Particularly in term-intensive fields such as science and mathematics, terminological differences caused serious comprehension and implementation problems. This situation rendered the academic foundation students had previously acquired in Turkish inaccessible in Arabic, pointing to a disjunction between their conversational abilities and the academic register demanded by the new medium of instruction.

Student S6 elaborated this situation as follows: “I learned science in Turkish, so things like ‘cell’ were easy, but now when they teach the same thing in Arabic, new words come up, and sometimes I don’t understand what they’re saying. For example, they said ‘nuvât,’ which turned out to mean ‘core’.” Similarly, S9 pointed out that differences in terminology in math class led to incorrect solutions: “The operation is the same in math, but the terms are different. When the teacher said ‘mütâvessit,’ I didn’t understand at first. Then I learned it meant average. But when I first understood the question, I solved it incorrectly.” S3 emphasized that technical terms create difficulties in written expression: “I had internalized some things in Turkish, so I didn’t struggle with written expression. But here, when writing Arabic paragraphs, I feel a lack of technical vocabulary. It’s as if my brain wants to think in Turkish, but I can’t find the words to translate.”

These findings indicate that academic language proficiency cannot be reduced to general language competence. Even students who are fluent in everyday Arabic experience a form of academic dislocation when technical terminology is reorganized in another language. This supports the view that academic language functions as a distinct register requiring explicit instructional scaffolding rather than implicit exposure.

#### Difficulty in conceptual transfer.

It was identified that linguistic differences did not remain only at the word level but also affected conceptual perception. Students emphasized that the mental concept maps they had constructed during Turkish-medium education lost their validity in Arabic-medium instruction.

Student S5 described this transition as follows: “I can understand the lesson, but sometimes I try to understand by thinking in Turkish. The teacher explains in Arabic, but I try to translate it into Turkish and understand it. This slows me down.” S11 elaborated the conceptual transfer problem in history class at a deeper level: “When I learn historical events in Arabic, it feels like I’m learning something else. In Türkiye, we called it the ‘War of Independence,’ but here they explain it differently. When the concepts change, my perception changes too.” S13 offered a different perspective: “I can speak Arabic, but the language of the school is different. The teacher says ‘hâl müzârî,’ a grammatical term. We didn’t learn such things in Turkish. It’s as if I’m relearning the same language at a different level.”

These accounts suggest that language transition can also entail a shift in epistemological framing. Conceptual transfer difficulties arise not only from linguistic gaps but from differences in pedagogical traditions and disciplinary discourse. As a result, students are required to reconstruct their knowledge schemas rather than merely translate existing ones.

#### Linguistic barriers in written production.

Students stated that they experienced serious problems in Arabic written expression and were constrained by a restrictive linguistic structure when expressing their thoughts. These barriers stem not only from insufficient linguistic knowledge but also from diminished self-confidence.

Student S2 explained the difficulty of the writing process as follows: “I find it very difficult to write compositions because it takes time to translate my thoughts into Arabic. I have thoughts, but I can’t form sentences. I’m always checking, ‘Is this correct?’” S10 explained the limitations experienced in exams: “I had to write in Arabic in the exam. I wrote, but it was incomplete because I don’t know how to use conjunctions or which tenses to use. If it were in Turkish, I could write a page.” Also, S1 stated: “At first, I had trouble constructing two sentences. Then I got some help. But I still can’t fully express myself in Arabic in writing. I can’t write my feelings.”

Written production emerged as one of the most fragile domains of academic adaptation. Concern about making linguistic mistakes can restrict students’ expressive capacity, turning writing into an anxiety-laden task. This highlights the dominance of written expression in Turkish rather than in Arabic, which points to the need for process-oriented writing support and formative feedback in students’ Arabic-medium schools.

Considered together, the three categories of terminological discontinuity, difficulty in conceptual transfer, and linguistic barriers in written production show that the difficulties returnee students face are concentrated in the academic register rather than in everyday communication. The terminological discontinuity reported by S6, who had learned science in Turkish and could no longer recognise the equivalent terms in Arabic, and by S9, for whom unfamiliar mathematical terms produced incorrect solutions, indicates that subject knowledge is bound to the language in which it was first acquired and does not transfer automatically. This is deepened in the second category, where S11’s sense that a historical event “feels like something else” in Arabic and S13’s encounter with the grammatical term hâl müzârî show that students must reconstruct their knowledge rather than simply translate it. The third category reveals where this strain becomes most visible: in writing, where S2’s constant self-checking and S10’s inability to use the right conjunctions and tenses turn composition into a hesitant, error-monitored task. Educationally, this suggests that returnee students are not simply lagging behind but are managing an additional cognitive demand that teachers may misinterpret as a lack of subject knowledge, since prior learning becomes temporarily inaccessible, rather than absent, when the medium of instruction changes. Sociolinguistically, these accounts indicate that the language of instruction operates as a gatekeeper to disciplinary knowledge: it is the gap between conversational fluency and academic language, most evident in the hesitant and self-correcting nature of students’ writing, that shapes the returnee experience and points to the need for explicit and sustained support in the academic register rather than an assumption of native competence.

### Theme 2: Social integration and academic confidence

Students who returned to Syria experienced difficulties not only linguistically but also socially and academically during reintegration. This theme was examined under three categories: teacher–student relationships, peer interactions, and academic self-confidence. Table 3 presents the categories, relevant codes, and sample extracts through which Theme 2 was inductively developed.

**Table 3:** Social integration and academic confidence.

Category	Codes	Sample Data Extract
**Difficulties in Establishing Relationships with Teachers**	Fear of speaking to teachers; formal communication; unclear expectations; lack of recognition.	“I am afraid I will make a mistake when talking to the teacher, so I hesitate to ask questions.” (S4)
**Lack of Peer Support**	Social isolation; exclusion from group work; lack of peer support; invisibility.	“When I entered the class, no one talked to me, even the student sitting next to me.” (S2)
**Decline in Academic Self-Confidence**	Loss of confidence; comparison with past success; sensitivity to evaluation; reduced motivation.	“I used to be successful, but now everyone looks at me as if I know nothing.” (S8)

#### Difficulties in establishing relationships with teachers.

Several students reported difficulties in communicating effectively with teachers due to linguistic and cultural differences. For example, S4 stated: “I’m afraid I’ll make a mistake when talking to the teacher. I used to speak Turkish comfortably, but here the teacher is very formal.” Similarly, S10 described a loss of recognition: “My teacher at the old school knew my name, but here they sometimes walk right past me.” On the other hand, S13 emphasized uncertainty regarding expectations: “Even though I know Arabic, I don’t understand what the teacher expects.”

These findings reveal that adaptation challenges extend beyond language to include shifts in power relations and pedagogical norms. The more formal and teacher-directed classroom interactions described by these participants may hinder help-seeking behaviours and weaken students’ sense of academic belonging.

#### Lack of peer support.

Peer relationships also emerged as a challenge. S2’s statement directly reflects the experience of social exclusion: “When I entered the new class, no one spoke to me. I thought they didn’t know me, but then they didn’t care. Even the child sitting next to me was talking to someone else.” Such social isolation may erode students’ sense of belonging and their motivation to engage in classroom activities. Furthermore, S7 noted their exclusion from collaborative learning environments, stating: “I had friends in Turkish class, and we worked together. Here, everyone keeps to themselves. When we have group work, no one includes me.” This statement indicates that the student is marginalized not only in terms of social integration but also in terms of pedagogical engagement, resulting in their disconnection from active learning methodologies. The fragility of peer relationships can have a detrimental impact on both academic and emotional well-being. S11 provided the following description of this situation in relation to daily life: “I even sit alone at meals. The first day was very difficult; no one showed me where to sit. That’s why I always enter the cafeteria last.”

Such statements show that peer exclusion intensifies the emotional cost of migration and reduces opportunities for informal learning. Therefore, integration policies implemented in schools after migration should not be limited to the language level; social and psychological adaptation must also be actively supported.

#### Decline in academic self-confidence.

Students reported a decline in academic self-confidence. This reflects a disconnect between students’ actual competencies and how these competencies are perceived. Without recognition and encouragement, previous academic success fails to translate into the new educational context. The feeling of not being academically competent is particularly evident when previous experiences of success are overshadowed. S1’s statement clearly illustrates this situation: “I feel like I fail in my classes here. I used to get very good grades in Turkish, but now the teacher thinks I don’t understand because I may not express myself better in Arabic concepts of the classes.” Students are forced to reconstruct not only the language but also the way they express themselves, which may negatively affect their self-confidence. S8 expressed the feeling that their academic competence is constantly being tested by their environment with the following words: “I used to be the best in the class, but now when I speak, everyone looks at me as if they are searching for mistakes. They act as if I know nothing.” This experience may undermine the student’s expectation of social approval and damage their academic self-confidence. Finally, S12 felt that their achievements were invisible to the teacher: “Even when I do my homework, it’s as if it’s not appreciated. The teacher doesn’t say anything when giving grades, but I can tell it from their face. That’s why I lose motivation while doing it.”

These examples illustrate that students encountering difficulties in their educational pursuits require not only the restoration of their academic performance but also the reconstruction of their sense of self-worth. Thus, the provision of social support at this juncture is of paramount importance.

Considered together, the three categories of difficulties in establishing relationships with teachers, lack of peer support, and decline in academic self-confidence show that reintegration is a social process as much as a linguistic one, and that academic confidence is a relational outcome rather than a fixed trait. The formality and distance students describe in their dealings with teachers, captured in S4’s fear of making a mistake when speaking and S10’s sense of no longer being recognised by name, restrict the help-seeking on which adaptation depends. At the peer level, the isolation reported by S2, who found that no one spoke to her, and by S11, who enters the cafeteria last because no one shows him where to sit, removes the informal interaction through which both language and belonging are ordinarily built. These social experiences feed directly into the third category: when prior competence goes unrecognised, students such as S1 and S8 begin to internalise an image of themselves as incapable that contradicts their actual knowledge, while S12’s sense that completed work goes unacknowledged erodes motivation further. Educationally, this indicates that confidence cannot be restored through language support alone. Sociolinguistically, these accounts reveal a process of misrecognition, in which a limited command of the academic register is read by teachers and peers as limited intelligence, so that a linguistic difficulty is socially reframed as a deficiency of the student. Restoring confidence therefore depends as much on changing how returnee students are positioned and perceived as on remediating their language.

### Theme 3: Re-adaptation to the education system

Participants described the transition from education in Türkiye to schooling in Syria as requiring not only linguistic adaptation, but also re-adaptation to pedagogical practices, curriculum content, and assessment practices. Accordingly, this theme focuses on curriculum differences, teaching methods, and assessment practices. Table 4 shows the relevant findings below.

**Table 4:** Re-adaptation to the education system.

Category	Codes	Sample Data Extract
**Curriculum Discontinuities**	Knowledge gaps; unfamiliar content; missing prerequisites; contextual mismatch.	“There are topics in the textbooks here that we never studied in Turkey.” (S3)
**Differences in Teaching Methods**	Teacher-centered instruction; reduced interaction; limited participation; fast pacing.	“In Türkiye we did experiments, but here the teacher just explains and moves on.” (S9)
**Challenges in Adapting to the Assessment System**	Unfamiliar exam formats; uncertainty about grading criteria; oral assessment uncertainty; perceived inconsistency in grading.	“I don’t know how oral grades are given, and that makes me nervous.” (S6)

#### Curriculum discontinuities.

The majority of students reported that the subjects they encountered after return did not align with the curriculum they had experienced in Türkiye and that they were therefore falling behind others academically. This difference is particularly evident in science and social studies classes. S3 clearly highlighted this problem with the following statements: “There are subjects in the textbooks here that I have never heard of. We didn’t cover these topics in Türkiye, and I fell behind, especially in science classes.” For these students, such gaps made it more difficult to understand the lessons and participate academically. S5 experienced a similar situation specifically in biology class: “I’m learning some things in biology for the first time here, whereas they were taught differently in Türkiye. I had never heard of some concepts.” This difference creates gaps not only in the level of knowledge but also in the conceptual framework. Especially in context-based subjects such as history, the fact that students have never encountered these topics before is a significant disadvantage. Supporting this, S13 stated: “In history class, the teacher explains the topic, but I don’t know the background because these topics were never covered in Türkiye before.”

These statements suggest that curricular discontinuities during post-migration reintegration can create cumulative learning gaps, particularly when returnee students encounter content or assumed prerequisites that were not part of their previous schooling, thereby placing them at a structural disadvantage within the specific school contexts they entered.

#### Differences in teaching methods.

Students often emphasized difficulties in moving from the more interactive teaching approaches they recalled in Türkiye to more teacher-directed practices in their current classrooms. S1’s statements highlight the pedagogical dimension of this transition: “In Türkiye, we did more activities, but here the teacher writes on the board and we copy it into our notebooks. It’s harder for me to enjoy the lessons.” For S1, this difference appeared to reduce enjoyment and opportunities for active participation in learning. S9 explained the effect of shifting from lab-based, hands-on learning to theoretical instruction as follows: “The lessons became very rote. In Turkey, we did experiments; here, they just explain and move on.” S9’s account similarly suggests that the shift from hands-on activities to explanation-based instruction reduced opportunities for active participation. S11 stated that the pace of the lessons was challenging for them: “Topics are covered quickly in math. In Türkiye, they were covered step by step, but here it’s as if everyone already knows.” S11’s account suggests that the pace of instruction did not fully accommodate this student’s adjustment needs.

In sum, students reported difficulty in adapting to the more teacher-directed instruction they described. More specifically, S1, S9, and S11 highlighted reduced interaction and rapid pacing. For these participants, reduced interaction and rapid pacing appeared to limit engagement and autonomy, suggesting the importance of instructional flexibility for diverse learner backgrounds.

#### Challenges in adapting to the assessment system.

Another area of adaptation is the differences in the measurement and evaluation system. Students expressed uncertainty about practices such as traditional exams, oral grades, and performance evaluations. S2 described the difference in assessment formats as follows: “I don’t know what kind of questions will be on the exam. In Türkiye, we had tests, but here there are traditional questions, and I can’t predict what I will write.” This uncertainty may make exam preparation and performance more difficult for returnee students. S6 described uncertainty about how oral grades were determined as follows: “We get oral grades here, but I don’t understand how they are given. Sometimes the teacher asks a question, but if we remain silent, we get a low grade.” S6’s account indicates that uncertainty about evaluation can itself become a source of pressure. Finally, S12 expressed confusion about the homework evaluation process: “Homework is graded, but I don’t know how it is evaluated. I got a different grade than someone who did the same homework, and I didn’t understand why.”

Taken together, these statements suggest that participants’ uncertainty about assessment criteria, feedback, and grading practices can affect their sense of control, academic confidence, and motivation. Transparent and formative assessment practices may therefore support re-adaptation.

Read across its three categories of curriculum discontinuities, differences in teaching methods, and challenges in adapting to the assessment system, Theme 3 shows that re-adaptation requires learning the implicit rules of an institution as much as mastering its formal content. The curriculum gaps students report, together with the shift toward the more teacher-directed classrooms participants described, place returnee students in school contexts whose expectations they have not been taught to read, while practices such as oral grading and open-ended written examinations carry tacit conventions that local students absorb over time but that remain opaque to newcomers. S2’s inability to predict the form of exam questions and S6’s confusion over how oral grades are assigned show that the difficulty lies less in the content itself than in the rules of participation, so that students may be penalised not for lacking knowledge but for lacking access to the system’s unwritten norms. Educationally, this reframes adaptation difficulty as a matter of institutional transparency rather than individual shortcoming, since their current school context may assume a shared background that returnee students do not possess. Sociolinguistically, the silence that earns a low oral grade is revealing: students withhold participation not out of disengagement but out of uncertainty about the discursive norms of the classroom, which demonstrates that the pedagogical and evaluative practices they encountered, and not language alone, can shape whether a returnee can show what they know.

### Theme 4: Psychosocial and contextual pressures

Syrian students who had returned after years of displacement described not only academic challenges but also psychosocial and contextual pressures affecting their school experiences. Based on the data, this theme was analysed in three categories: emotional strain and disrupted belonging, concentration and emotional difficulties in learning, and increased family responsibilities. Table 5 shows the categories, codes, and sample extracts below.

**Table 5:** Psychosocial and contextual pressures.

Category	Codes	Sample Data Extract
**Emotional Strain and Disrupted Belonging**	Alienation; emotional exhaustion; loss of belonging; disrupted familiarity.	“I always feel like a foreigner in the classroom.” (S6)
**Concentration and Emotional Difficulties in Learning**	Concentration problems; emotional distress; reduced motivation; sensitivity to classroom interactions.	“Even when I come to school, my mind is somewhere else and I cannot focus.” (S3)
**Increased Family Responsibilities**	Caregiving duties; household labor; economic contribution; irregular attendance.	“After school I take care of my siblings, so I don’t have time to study.” (S1)

#### Emotional strain and disrupted belonging.

Although the students voluntarily returned to Syria after living in Türkiye for a long time, they described emotional strain associated with return. For example, S4 expressed that this return created emotional exhaustion and a feeling of social exclusion with the following words: “Coming back here exhausted me. It was also difficult to get used to school because the children here see us as different.” This statement shows how fragile not only academic but also social integration is. The students’ feeling of being “foreign” indicates a weakening of their sense of belonging. S6 described the emotional exclusion they experienced in the classroom as follows: “I always feel like a foreigner here. When I enter the classroom, it feels like everyone is looking at me.” This sense of alienation may undermine students’ participation and self-confidence. S10, on the other hand, conveys the temporal impact of war and migration: “We left because of the war, now we’re back, but everything has changed so much. I don’t feel the same here anymore.” This account suggests a loss of belonging that is not only spatial but also temporal, as a previously familiar place is experienced as changed after return.

Across these sample extracts, S4, S6, and S10 described feelings of exhaustion, alienation, and loss of belonging. Taken together, these accounts suggest that re-migration can disrupt emotional continuity, producing a sense of “double displacement” that may affect school adaptation.

#### Concentration and emotional difficulties in learning.

Many students described mental fatigue, concentration difficulties, and reduced motivation in relation to their experiences of war, migration, and return. For instance, S3 directly linked this situation to the learning process: “Even if I come to school, I can’t listen to the lessons. My mind is elsewhere, as if I can’t learn anything.” This account indicates concentration difficulty in the context of return and prior displacement, but the present data do not establish a trauma-specific mechanism. S8 described emotional distress in response to raised voices in the classroom: “Sometimes I want to cry in class. I feel especially bad when voices are raised.” This statement shows that particular classroom interactions were emotionally difficult for this student; without trauma-specific assessment, however, the response cannot be interpreted diagnostically. S11 also linked academic failure to self-esteem: “When I fail an exam, I immediately feel sad. I wasn’t like this before. Now I have no confidence in myself.”

In this context, academic failure may further undermine self-esteem for students already experiencing emotional strain. Because the study did not employ a trauma-specific instrument or clinical assessment, these accounts are interpreted as psychosocial strain and concentration difficulties rather than as evidence of trauma responses, triggers, or weakened psychological resilience. Psychologically safe and responsive educational environments may therefore be valuable.

#### Increased family responsibilities.

After migration, many students stated that they were unable to focus sufficiently on their academic studies due to increased responsibilities at home. S1 described how she spends her time outside of school caring for her siblings: “My mother works, so I take care of my little brother. When I leave school, I go straight home and cook dinner.” This quote shows how caregiving duties can conflict with study time. On the other hand, S7 touched upon the direct impact of housework on school success: “I have a lot of responsibilities at home. I can’t study because I have to tidy up the house.” For these students, domestic or economic responsibilities can reduce the time available for education. S13’s statement highlights the economic aspect of this situation: “My father is unemployed, sometimes I work at the street market. There are days when I can’t go to school, and then I fall behind in my studies.” These excerpts show that some returnee students were managing responsibilities extending beyond schooling and, in some cases, assuming adult-like roles at an early age. In other words, increased domestic and economic burdens position education as secondary, particularly for vulnerable students. This highlights the intersection of migration, poverty, and educational inequality.

These thematic findings demonstrate that post-war re-migration affects students’ academic trajectories through intertwined linguistic, pedagogical, social, and psychosocial mechanisms. Effective educational responses must therefore be holistic, integrating language support, social inclusion strategies, psychosocially responsive practices, and curriculum alignment policies.

Taken together, the three categories of emotional strain and disrupted belonging, concentration and emotional difficulties in learning, and increased family responsibilities show that the academic struggles of returnee students cannot be separated from the emotional and material conditions of their lives after return. The sense of alienation conveyed by S6, who feels like a stranger in the classroom, and the concentration problems students describe indicate that psychosocial pressures associated with displacement can enter the learning process, so that difficulty in studying may reflect emotional strain and contextual pressures rather than a lack of effort. These pressures are compounded by external ones: when a student misses school to work at a market, as S13 describes, or studies only after caring for younger siblings and managing the household, as S1 and S7 report, the classroom difficulties documented in the earlier themes are intensified by responsibilities that schooling may not fully take into account. Educationally, this suggests that language and curriculum interventions, however carefully designed, may achieve less if they ignore the out-of-school realities that determine whether a child can attend, concentrate, and continue. These accounts also show that domestic and economic responsibilities were not distributed uniformly across participants, underscoring the need to consider how displacement, poverty, and family precarity shape educational participation.

## Discussion

The present study illuminates the complex re-adaptation trajectories of Syrian students transitioning from Turkish-medium instruction back to Arabic-medium schooling in Syria. By framing this process as a “reverse academic transition,” the findings challenge the prevailing focus in refugee education on host-country adaptation [10], shifting the analytical lens to the under-explored dynamics of return [4]. The data reveal that re-adaptation is not merely a restoration of a native language but a multi-layered negotiation of linguistic registers, pedagogical cultures, and psychosocial ecologies. What emerges from the participants’ narratives is a picture of students caught between two educational worlds, struggling to reconcile their past learning experiences with present institutional demands.

A primary contribution of this study is the distinction between conversational fluency and academic proficiency evident in the finding of ‘Terminological Discontinuity’. While students demonstrate competence in colloquial Arabic, often acquired through family interactions, they face a critical gap when confronted with specialized vocabulary of mathematics, science, or social studies classes [5,8,17]. This is not trivial. The reported ‘Difficulty in Conceptual Transfer’ reveals that when students learned photosynthesis or algebraic equations in Turkish, these concepts became cognitively bound to Turkish terminology and explanatory frameworks. Transferring these understandings into Arabic is not simple translation; it requires cognitive restructuring and construction of new semantic networks [41]. The fragility observed in ‘Linguistic Barriers in Written Production’ further confirms that literacy and formal registers are the first domains to suffer under reduced input conditions [7,21]. Consequently, the return experience should be interpreted not as a return to the known, but as a confrontation with an academic language gap requiring explicit bridging pedagogy rather than an assumption of native competence [14]. Recent evidence from other return-migration settings reinforces this interpretation: studies of returnee families indicate that children’s linguistic re-socialisation into the home-country school language is rarely automatic and must be actively negotiated by families and schools alike [42]. Viewed in this light, the participants’ experiences are not an idiosyncratic by-product of the Syrian context but an instance of a broader sociolinguistic regularity in which the language of the “homeland” confronts returnee children as a school language they have yet to master.

However, linguistic challenges alone do not explain the depth of students’ struggles. The finding of ‘Lack of Peer Support’ and fear of corrective feedback illustrates how interactional exclusion restricts the comprehensible output necessary for language development [30]. Consistent with sociocultural theories [31,43], students’ academic confidence was closely linked in their accounts to the quality of teacher-student interactions and peer inclusion, yet several described feeling isolated during group work, watching classmates collaborate effortlessly while they struggled to follow discussions. This social marginalization creates a vicious cycle. That is, reduced interaction leads to slower language development, which reinforces social exclusion. Research distinguishes teacher-centred from student-centred approaches, with the former limiting interaction and student agency [44], while multilingual and translanguaging pedagogies aim to scaffold participation for students learning in an additional language [45]. The shift captured in ‘Differences in Teaching Methods’, from the more interactive routines participants recalled in their Turkish schools to the more teacher-directed practices they described in their current Syrian classrooms, appeared to exacerbate these barriers, creating a double binding where students must simultaneously decode a new instructional culture while lacking the linguistic scaffold to participate meaningfully. Comparable patterns have been documented among returnee pupils in other national contexts, where teachers were often unprepared for children re-entering the system and tended to frame limited proficiency in the school language as an obstacle rather than a workable starting point [46]. Recent large-scale evidence further shows that close teacher–student relationships significantly improve returned migrant children’s academic adjustment by strengthening school attitudes and resilience [47]. Taken together, these findings suggest that the relational climate of the classroom is not a peripheral comfort but a core mechanism of post-return academic recovery. This relational interpretation is also consistent with Rind’s structure-agency perspective, which conceptualizes learning experiences through the interaction of individual agency, classroom community, and institutional influences rather than learner characteristics alone [48]. Recent evidence further indicates that teacher-child and peer relationship quality are positively associated with school belonging among immigrant, refugee, and non-immigrant children, while supportive teacher relationships can moderate some outcomes associated with proficiency in the language of schooling [49].

Beyond interpersonal dynamics, participants also described curricular and assessment factors that compounded these challenges. The ‘Curriculum Discontinuities’ and ‘Challenges in Adapting to the Assessment System’ reveal that when curricula do not align and foundational concepts assumed in Syria were never covered in Türkiye, then students face not merely a language barrier but a content knowledge barrier [18]. Grading criteria and oral assessment practices that participants perceived as unclear add another layer, with students reporting uncertainty about what constitutes a good answer, creating persistent anxiety that undermines academic confidence. In this context, applying monolingual assessment norms to students with bilingual schooling histories risks pathologizing their adaptation process, framing what is essentially a transitional challenge as individual failure. This supports the argument that equitable reintegration requires flexible, formative, and background-sensitive assessment frameworks recognizing the latent bilingualism of returnee students [50].

Yet even when linguistic, social, and structural barriers are addressed, profound psychosocial dimensions evident in ‘Emotional Strain and Disrupted Belonging’ and ‘Concentration and Emotional Difficulties in Learning’ cannot be ignored. The reported concentration difficulties and emotional exhaustion should not be reduced to individual deficits; rather, they can be interpreted in relation to the cumulative pressures of re-migration and post-conflict daily stressors [9,10]. Furthermore, ‘Increased Family Responsibilities’, which included caregiving for younger siblings or contributing to family income, directly constrain the time, energy, and mental space available for studying [51]. When a student must work in the afternoon to help support the family, attending extra tutoring becomes impossible. When a student is managing emotional strain associated with displacement and family pressures, concentrating during a mathematics class may become considerably more difficult. This holistic understanding underscores that educational interventions cannot succeed in isolation from psychosocial support. This interpretation aligns with recent research on remigrant pupils, which characterises return as an experience with high vulnerabilising potential that elevates stress and complicates educational reintegration, particularly for children who were not prepared for the move [52]. Read through this lens, the psychosocial burden observed in the present study appears constitutive of the reverse academic transition itself rather than incidental to it. This interpretation is consistent with recent evidence suggesting that positive and potentially protective factors, including resilience, prosocial behaviour, and school belonging, may be more closely associated with migrant students’ self-reported executive functioning than the set of stress-related factors examined, supporting a resource-oriented rather than exclusively deficit-focused perspective [53].

### Limitations and future research

Several limitations should be considered when interpreting the findings of this study. First, the study was conducted with a relatively small sample of 15 participants. Given the exploratory qualitative design and its emphasis on depth rather than breadth, the findings should be interpreted as context-specific insights rather than being broadly representative of all Syrian returnee students.

Second, the participants were between 11 and 14 years of age. While middle-school students are capable of describing their educational experiences and challenges, older adolescents or adult participants may provide more extensive and reflective accounts of language transition, educational adaptation, and identity-related experiences.

Third, data were collected through written online responses rather than face-to-face interviews. Although this approach enabled access to participants who had returned to Syria and were geographically inaccessible, it limited opportunities for follow-up questions, clarification, and deeper probing of participants’ responses. Consequently, some experiences may not have been explored in the same depth that could be achieved through interviews or focus groups.

Finally, the study focused on Syrian students who had experienced a specific educational trajectory involving migration to Türkiye and subsequent return to Syria. Therefore, the findings reflect a particular sociocultural and educational context and may not be transferable to all returnee students or other migration settings.

Future research could expand this line of inquiry by involving larger and more diverse participant groups from different educational contexts. Researchers may also employ triangulated data collection methods, such as interviews, classroom observations, teacher perspectives, and parent perspectives, to obtain a more comprehensive understanding of students’ adaptation experiences. In addition, longitudinal studies following students over extended periods may provide deeper insights into how academic, linguistic, and psychosocial adaptation develops after transitioning between educational systems.

## Conclusion

This study suggests that the return of refugee students to their country of origin may constitute a distinct educational phase requiring targeted intervention. Among the participants of this study, the ‘reverse academic transition’ was characterized by a fundamental mismatch between students’ linguistic repertoires, which were shaped by years of Turkish-medium instruction, and institutional expectations in Arabic-medium Syrian schools manifesting across linguistic, pedagogical, structural, and psychosocial dimensions.

While these findings are exploratory and context-specific, they point to several implications for policy and practice. Potential steps in such contexts include implementing academic Arabic support programs targeting disciplinary terminology and academic writing; adopting psychosocially responsive and interaction-based teaching strategies to rebuild academic confidence and peer networks; and integrating psychosocial support with flexible schooling arrangements to mitigate the impact of economic duties and family stressors. Ultimately, successful re-adaptation depends on moving beyond the binary of refugee versus citizen to acknowledge the hybrid educational identities formed in displacement. Syrian returnees occupy a unique position, carrying valuable linguistic and cultural resources alongside specific challenges, and designing educational responses that build on students’ strengths while addressing their needs represents the pathway toward truly equitable and effective reintegration. Given the qualitative design and the small, context-specific sample, these conclusions should be read as preliminary insights into the experiences of a particular group of returnee students rather than as broadly generalizable claims.

### Recommendations for policy and practice

Building on the identified concept of reverse academic transition, the following recommendations emerge as potential pathways toward equitable re-adaptation of returnee students to Arabic-medium schooling, moving beyond ad-hoc solutions toward structural, evidence-based interventions.

Rather than general language remediation, re-adaptation efforts should prioritize specialized Academic Arabic bridging curricula targeting the academic register gap through discipline-specific terminology and advanced literacy skills [5,8]. These interventions should be guided by individualized language transition maps acknowledging that students need not relearn content but rather acquire the linguistic tools to access and demonstrate their existing knowledge. To mitigate the cognitive load of conceptual transfer, educational materials should leverage students’ prior linguistic assets through bilingual instructional scaffolding, such as glossaries, visual aids, and comparative conceptual guides linking Turkish and Arabic terms, respecting the cognitive reality that academic concepts are stored language-specifically [41].

Given the intersection of migration-related and daily reintegration stressors revealed in findings of concentration difficulties and emotional exhaustion, psychosocial support must be embedded into school culture rather than treated as a peripheral service. Schools should implement psychosocially responsive pedagogy that recognizes students’ reported difficulties not as individual deficits but in relation to displacement and current contextual pressures [9,10]. Simultaneously, teachers require specialized professional development emphasizing greater instructional flexibility and opportunities for interactive, student-centred learning that create opportunities for meaningful participation; necessary conditions for language development among students who feel alienated by more teacher-directed classroom interactions [30,31].

Finally, policy frameworks should recognize returnee students as a distinct demographic whose needs fit neither the standard native profile nor the traditional refugee category. Policy frameworks in the Syrian context could consider provisions for this group within longer-term integration strategies with flexible assessment regulations that distinguish between language proficiency and content knowledge [50]. Successful re-adaptation is likely to require systemic recognition that these students embody hybrid educational identities formed in displacement, identities that represent not deficits to be remediated but valuable resources to be acknowledged within a truly inclusive educational vision.
