# Supplementary material for: The hidden challenges of return migration: Linguistic and academic re-adaptation of Syrian students to the Arabic Education System
Source: PLoS One. 2026 Oct 16;21(10):e0361042. doi: 10.1371/journal.pone.0361042 (PMC13674759; doi:10.1371/journal.pone.0361042)
Supplement: Supplementary file 2 [file pone.0361042.s003.docx]

**RESPONSE TO THE ACADEMIC EDITOR AND REVIEWERS**

**Manuscript ID: PONE-D-26-11047R1**

**Manuscript Title: The Hidden Challenges of Return Migration: Linguistic and Academic Re-Adaptation of Syrian Students to the Arabic Education System**

**Dear Academic Editor and Reviewers,**

We sincerely thank the Academic Editor and the reviewers for their careful second-round evaluation of our manuscript. We are grateful that the substantial improvements made in the previous revision were recognized, and we have addressed each remaining point carefully. In this revision, we refined the methodological description, removed terminology that could imply unsupported conclusions, clarified the sampling and coding chronology, strengthened the contextual and relational interpretation of the findings, added relevant recent literature, and completed a manuscript-wide language and terminology review. All manuscript changes are shown in the tracked-changes version. Below, each reviewer comment is reproduced verbatim, followed by our response and the location of the corresponding revision.

**Response to the Academic Editor**

**Editor comment:** Decision: Minor Revision. Both reviewers consider the manuscript interesting, relevant, and methodologically sound, and they recognize its potential contribution to the literature on refugee education and the educational consequences of return migration. Overall, the manuscript has been substantially improved. However, both reviewers have identified several minor issues that should be addressed before the manuscript can be accepted. In particular, the authors are requested to:

**Response:** We thank the Academic Editor for the encouraging assessment and for identifying the remaining issues so clearly. We addressed each of the five requested areas as detailed below and in our point-by-point responses to Reviewers #1 and #5.

**Editor request 1:** Provide a clearer justification for the phenomenological design and explain how phenomenological principles informed the data collection and analysis.

**Response:** After reconsidering the methodological fit in light of the reviewer’s concern, we agreed that retaining a phenomenological label would imply a more specific methodological commitment than was warranted by the written questionnaire and qualitative content-analysis procedures. Rather than adding a retrospective phenomenological justification, we removed the phenomenological designation throughout and now describe the study consistently as an exploratory qualitative study employing qualitative content analysis.

**Changes in manuscript:** Abstract; Methods; Research Design; Participants; and Limitations and Future Research. The Methods now state, for example, “An exploratory qualitative approach was adopted,” and describe the analytic procedure as qualitative content analysis.

**Editor request 2:** Revise potentially diagnostic or overly strong psychological terminology and adopt a more contextual and relational interpretation of students’ experiences.

**Response:** We revised the manuscript throughout to distinguish participants’ reported experiences from psychological interpretation and removed wording that could be read as diagnostic. Theme 4 was reframed around psychosocial and contextual pressures, and we added explicit methodological caution where concentration and emotional difficulties are discussed. We also strengthened the relational/contextual interpretation using Rind (2016) and recent resource-oriented evidence from Opaas et al. (2024).

**Changes in manuscript:** Abstract; Findings (Theme 4); Discussion; Conclusion; and Recommendations for policy and practice. Theme 4 is now titled “Psychosocial and contextual pressures,” with the categories “emotional strain and disrupted belonging,” “concentration and emotional difficulties in learning,” and “increased family responsibilities.”

**Editor request 3:** Qualify broad statements about Turkish and Syrian educational systems so that interpretations remain grounded in the participants’ accounts.

**Response:** We systematically narrowed system-level claims to the evidential level supported by the data. Statements about pedagogy, curriculum, assessment, and classroom practice are now framed as participants’ perceptions and experiences in their previous and current schools rather than as general descriptions of two national education systems.

**Changes in manuscript:** Abstract; Research Question 3; Findings (Theme 3); Discussion; Conclusion; and Recommendations. Research Question 3 now asks about “the curricular, pedagogical, and assessment practices they experience in their post-return schools.”

**Editor request 4:** Clarify the procedures and justification concerning data saturation, coding, and the development of the coding framework, and reconcile the relevant tables and supplementary materials.

**Response:** We clarified the chronology of recruitment, data collection, and coding and removed the earlier prospective-saturation interpretation. The final sample reflected eligible students who were accessible and voluntarily participated during the predefined recruitment period; recruitment was not terminated on the basis of saturation. Coding began only after the data-collection period had ended and the full set of 15 responses was available. We also clarified the analytic hierarchy from meaningful units to initial codes, categories, and four overarching themes. Because saturation was not used prospectively to determine or terminate recruitment, we removed the saturation claim and will not resubmit the previous participant/code and saturation tables as supporting information; retaining a table labelled as saturation could imply a procedure that was not used prospectively. The main manuscript’s substantive thematic tables remain Tables 2–5.

**Changes in manuscript:** Methods (Participants and Data Analysis); Findings (Tables 2–5). The previous Code_and_Saturation_Tables supporting file is removed from the R2 submission package.

**Editor request 5:** Carefully proofread the manuscript for grammatical and typographical errors, ensure consistent terminology, and strengthen the discussion by engaging with a few relevant recent studies, including those suggested by the reviewers.

**Response:** We completed a manuscript-wide proofreading and terminology-consistency review in direct response to this request and Reviewer #1. We standardized the description of the data-collection instrument as an open-ended written questionnaire, corrected residual wording and terminology inconsistencies, and aligned terms such as academic confidence and re-adaptation across sections. We also strengthened the Discussion with two additional recent empirical studies, Ialuna et al. (2024) and Opaas et al. (2024), and incorporated Rind (2016) where it directly supports the requested relational/contextual interpretation.

**Changes in manuscript:** Throughout the manuscript, especially Methods, Findings, Discussion, and References. Three references were added to the reference list: Rind (2016) [51], Ialuna et al. (2024) [52], and Opaas et al. (2024) [53].

**Response to Reviewer #1**

**Comment:** The manuscript addresses an important and underexplored topic concerning the educational reintegration of voluntarily returned Syrian students. The study is well designed, the methodology is appropriate, and the findings provide meaningful theoretical and practical contributions to refugee education and bilingual learning research. The discussion is coherent and the implications are clearly articulated. However, a few minor revisions are recommended before acceptance. The authors should carefully proofread the manuscript for minor grammatical and typographical errors, ensure consistent terminology throughout the text, and strengthen the discussion by explicitly linking the findings to a few additional recent studies. Subject to these minor corrections, the manuscript is suitable for publication.

**Response:** We sincerely thank the reviewer for the positive assessment and for identifying these final improvements. We conducted a manuscript-wide proofreading and terminology-consistency review rather than limiting the revision to isolated corrections. Among other refinements, we standardized the description of the data-collection instrument as an “open-ended written questionnaire,” corrected an incorrect reference to interviews in the ethics section so that it now states that the students provided verbal assent before completing the questionnaire, used “academic confidence” consistently when referring to confidence rather than self-efficacy, and corrected minor grammatical, typographical, and wording inconsistencies throughout the manuscript. Figure 1 was also revised to ensure full consistency with the terminology used in the Findings. In particular, the figure now uses the revised labels “Difficulties in academic language adaptation,” “Terminological Discontinuity,” “Curriculum Discontinuities,” “Decline in Academic Self-Confidence,” and “Psychosocial and contextual pressures,” together with the revised psychosocial categories “Emotional Strain and Disrupted Belonging” and “Concentration and Emotional Difficulties in Learning.” We also strengthened the Discussion with two additional recent empirical studies: Ialuna et al. (2024), which provides recent evidence on teacher/peer relationships, school belonging, and language-of-schooling challenges among immigrant and refugee children, and Opaas et al. (2024), which supports a resource-oriented interpretation of migrant students’ functioning. Rind (2016) was additionally incorporated in response to Reviewer #5’s request for a relational and contextual perspective.

**Changes in manuscript:** Manuscript-wide proofreading and terminology review; Figure 1, Discussion paragraphs addressing relational school adjustment and psychosocial/contextual pressures; References [51]–[53].

**Response to Reviewer #3**

**Comment:** I have no further Comments. All my comments have been addressed and I am happy with the end results of the paper.

**Response:** We sincerely thank the reviewer for the encouraging evaluation of the manuscript and for confirming that the previous comments have been fully addressed. We are grateful for the reviewer’s contributions across the review process, which helped improve the clarity and transparency of the manuscript.

**Response to Reviewer #5**

**General comment:** This is an interesting paper that shifts attention from refugee integration in host countries to the comparatively neglected educational consequences of return migration, particularly the transition from Turkish-medium to Arabic-medium schooling. The revision has clearly improved the paper, especially in its articulation of the research questions, acknowledgement of limitations, expansion of the theoretical discussion, and description of trustworthiness procedures. However, I still see some issues that should be addressed before publication. I have specifies these issues bellow:

**Response:** We thank the reviewer for the careful second-round reading and for recognizing the improvements made in the previous revision. We found the remaining methodological and interpretive concerns constructive and have addressed each one below. In particular, we reconsidered the design label rather than defending it retrospectively, narrowed psychological and system-level inferences, clarified the sampling/coding chronology, and strengthened the contextual interpretation of students’ experiences.

**Comment 1:** The manuscript identifies the study as phenomenological, yet the empirical material was generated through seven open-ended questions administered through Google Forms to 15 students aged 11–14. Although the authors appropriately explain that geographical constraints prevented face-to-face interviews, written responses collected within approximately 15–25 minutes necessarily provide limited opportunities for probing, clarification, and deeper exploration of lived experience. The authors should therefore provide a more convincing explanation of what specifically makes the study phenomenological rather than simply an exploratory qualitative study employing qualitative content analysis. This should include identification of the particular phenomenological orientation adopted and a clearer explanation of how phenomenological principles informed both data collection and interpretation. The authors may find it useful to engage with empirical examples demonstrating how phenomenological inquiry can connect participants’ lived experiences, contextual conditions, and meaning-making processes while maintaining methodological coherence; for example, Soomro et al. (2025) may provide a relevant methodological point of comparison in the field of ESL and education.

**Response:** We agree with the reviewer’s methodological concern. After reconsidering the relationship among our research questions, written data-collection procedure, and analytic strategy, we concluded that the phenomenological label was more specific than our procedures warranted. We therefore chose not to add a retrospective phenomenological orientation or to defend the label on the basis of participants’ lived experiences alone. Instead, we removed all phenomenological terminology and now describe the study consistently as an exploratory qualitative study using qualitative content analysis. The Research Design section explains that the aim is an in-depth, context-sensitive understanding of students’ experiences and that qualitative content analysis is the analytic procedure used to organize and interpret the written narratives. The Limitation section continues to acknowledge explicitly that written online responses did not permit the depth of follow-up probing available in interviews. We also carefully evaluated Soomro et al. (2025), as suggested. Because the revised manuscript no longer characterizes the design as phenomenological, we did not use this work as methodological support for a design label that we have removed.

**Changes in manuscript:** Abstract: “Using an exploratory qualitative design…” Methods, Research Design: “An exploratory qualitative approach was adopted.” The subsequent design paragraphs now explain the exploratory, context-sensitive purpose and the role of qualitative content analysis. All phenomenological terminology was removed from the manuscript, including the design and limitation statements.

**Comment 2:** The manuscript sometimes uses terminology that approaches psychological diagnosis despite the absence of a trauma-specific instrument, clinical assessment, or in-depth psychological interviews. Statements describing concentration difficulties as a “classic effect of trauma,” raised voices as trauma “triggers,” or students as having weakened psychological resilience go beyond what can confidently be inferred from short written accounts. The authors should distinguish more carefully between what participants actually reported and the researchers’ interpretation through the trauma literature. Terms such as “migration-related distress,” “psychosocial strain,” or “experiences potentially consistent with trauma-related responses” would be more defensible. More broadly, the paper would benefit from adopting a relational and contextual interpretation of students’ experiences rather than treating their responses primarily as individual psychological conditions. In this respect, the authors may consider the structure–agency perspective developed by Rind (2016), which emphasises how learners’ experiences emerge through interactions among individual agency, institutional structures, social relationships, and contextual constraints.emerge through interactions among individual agency, institutional structures, social relationships, and contextual constraints. Such a perspective could complement the manuscript’s existing argument that returnees’ difficulties are produced partly through institutional mismatches rather than individual deficits.

**Response:** We agree and revised this part of the manuscript extensively but selectively, preserving the participant accounts while narrowing the interpretive claims. We removed wording such as “classic effect of trauma,” “triggers,” “deep psychological scars,” and “weakened psychological resilience,” and we no longer infer trauma-specific mechanisms from the written responses. Theme 4 has been reframed as “Psychosocial and contextual pressures.” These terminology changes were also incorporated into Fig. 1 so that the visual representation of the thematic framework fully matches the revised Findings section. The revised Findings explicitly state, for example, that one participant’s account indicates concentration difficulty “but the present data do not establish a trauma-specific mechanism,” and that another participant’s response to raised voices “cannot be interpreted diagnostically” without trauma-specific assessment. We then state explicitly that these accounts are interpreted as psychosocial strain and concentration difficulties rather than as evidence of trauma responses, triggers, or weakened psychological resilience. We also adopted the reviewer’s broader relational/contextual recommendation. Rind (2016) has been incorporated into the Discussion as a conceptual bridge showing how learning experiences can be understood through the interaction of individual agency, classroom community, and institutional influences rather than learner characteristics alone. To strengthen this interpretation with recent empirical evidence, we also added Opaas et al. (2024), whose findings support a resource-oriented perspective that attends to school belonging, resilience, prosocial functioning, and current social conditions alongside stress-related factors.

**Changes in manuscript:** Abstract; Findings, Figure 1, Theme 4 (“Psychosocial and contextual pressures”); Discussion; Conclusion; Recommendations for policy and practice; References [51] and [53].

**Comment 3:** Several sections move from students’ individual accounts to rather broad characterisations of Turkish schooling as student-centred and interactive and Syrian schooling as teacher-centred, traditional, transmission-oriented, or authoritarian. For instance, students’ experiences of copying from the board, limited experimentation, and rapid instructional pacing are interpreted as evidence of a broader transition between contrasting pedagogical systems. The data support claims about how these particular students perceived differences between their previous and current classrooms, but a sample of 15 students cannot support general statements about two national education systems. The authors should therefore consistently qualify these interpretations—for example, “participants perceived their current classrooms as more teacher-centred than the classrooms they had previously experienced in Türkiye.” Similar caution is required when discussing curriculum quality, assessment transparency, teacher authority, and pedagogical culture.

**Response:** We agree with this distinction and have revised the manuscript so that the level of inference matches the level of evidence. We no longer characterize Turkish or Syrian education systems broadly on the basis of the 15 participants’ accounts. Instead, statements about pedagogy, curriculum, assessment, teacher practices, and classroom interaction are explicitly tied to what participants recalled or experienced in their particular previous and current schools. We also revised Research Question 3 so that it no longer presupposes national-system characteristics and instead asks about the curricular, pedagogical, and assessment practices students experience in their post-return schools. For example, the Abstract now refers to “pedagogical practices that participants perceived as more teacher-directed” and “assessment practices that they perceived as unclear.” In the Discussion, the previous cross-system characterization is now framed as a shift “from the more interactive routines participants recalled in their Turkish schools to the more teacher-directed practices they described in their current Syrian classrooms.” Assessment claims are likewise framed as grading criteria and oral assessment practices “that participants perceived as unclear.” These revisions preserve the substantive finding while keeping the interpretation grounded in participants’ accounts.

**Changes in manuscript:** Abstract; Introduction, Research Question 3; Findings, Theme 3; Discussion; Conclusion; Recommendations for policy and practice.

**Comment 4:** The response to reviewers states that all twelve sub-codes had emerged by the fifth participant and that no new codes appeared subsequently, which is interpreted as evidence that saturation had been achieved. This claim requires further methodological justification. The authors should explain the order in which responses were collected and analysed, whether coding occurred concurrently with recruitment, how saturation was operationally defined, and whether the sample size was predetermined. In a fixed dataset analysed retrospectively, observing that later participants generated no new codes is not necessarily equivalent to demonstrating prospective theoretical or meaning saturation. Moreover, there appears to be inconsistency between the response letter—which indicates that dedicated participant/code and saturation tables were added—and the clean manuscript, where Tables 2–5 represent the four substantive themes. The authors should reconcile the table numbering and provide the coding/saturation material as supplementary information if necessary. Importantly, all participant-by-code entries must derive directly from the underlying data rather than being retrospectively completed to create a more symmetrical coding matrix.

Rind, I. A. (2016). Conceptualizing students’ learning experiences in English as a second language in higher education from structure and agency. Cogent Social Sciences, 2, 1191978.

Soomro, R. B. K., Khan, R. A., & Rind, I. A. (2025). Exploring ESL teachers’ experiences about ICT-based 4Cs skills: A phenomenological study. Discover Education, 4(12).

**Response:** We thank the reviewer for drawing this important distinction between a retrospective pattern in a fixed dataset and prospective saturation used to determine recruitment. The coding-progression and saturation material was added in the previous revision in direct response to a reviewer request for greater transparency regarding how the coding developed across participants. The present comment made clear, however, that describing the later absence of new codes as “data saturation” could imply that saturation was used prospectively to determine sample adequacy or to stop recruitment. That was not the procedure used in this study. We have therefore revised the methodological reporting rather than attempting to strengthen a saturation claim that does not describe the recruitment process. The Participants section now clarifies that the final sample reflected eligible students who were accessible and voluntarily participated during the predefined recruitment period and that recruitment was not terminated on the basis of prospective saturation. The Data Analysis section now states that coding commenced only after the data-collection period had ended and the complete set of 15 responses was available; coding therefore did not occur concurrently with participant recruitment. All 15 responses were analysed in a fixed analytic sequence, and that sequence played no role in determining recruitment or sample size. We have removed the statement that saturation was reached by the fifth participant. We also reconciled the table issue. The main manuscript’s Tables 2–5 remain the four substantive thematic tables. Because prospective saturation was not used to determine recruitment, we do not resubmit the previous participant/code and saturation tables as supplementary information. This avoids retaining a “saturation” representation that could imply a prospective sampling procedure that was not used. Transparency regarding the coding framework is instead provided directly in the revised Data Analysis section, which now describes the progression from meaningful units to initial codes, categories, and four overarching themes, together with the thematic tables and representative participant extracts in the Findings. This revision changes the methodological reporting only; it does not alter the dataset, coding, themes, or substantive findings.

**Changes in manuscript:** Methods, Participants and Data Analysis. The prospective saturation claim was removed. The previous Code_and_Saturation_Tables supporting file will not be resubmitted. Main-text Tables 2–5 remain the four substantive theme tables.

**Reference-list changes in this revision**

**As requested in the decision letter, we note the following additions to the reference list:**

[51] Rind IA. Conceptualizing students’ learning experiences in English as second language in higher education from structure and agency. Cogent Soc Sci. 2016;2(1):1191978. doi: 10.1080/23311886.2016.1191978.

[52] Ialuna F, Civitillo S, McElvany N, Leyendecker B, Jugert P. Resilience in multicultural classrooms: School relationships can protect the school adjustment of immigrant, refugee and non-immigrant children. Br J Educ Psychol. 2024;94(4):1271-93. doi: 10.1111/bjep.12713.

[53] Opaas M, Wentzel-Larsen T, Nissen A, Hilden PK, Punamäki RL, Spaas C, et al. Migrant students’ self-reported executive function skills in relation to mental health, postmigration stress, and positive appraisals of self and the social context. Eur J Psychotraumatol. 2024;15(1):2430923. doi: 10.1080/20008066.2024.2430923.

**Regarding the reviewer-suggested Soomro et al. (2025) reference:** we evaluated the study as suggested. Because the revised manuscript no longer identifies the design as phenomenological, we did not cite it as methodological support for a phenomenological orientation that has been removed.

We thank the Academic Editor and the reviewers once again for their careful and constructive feedback. We believe that the revisions have resolved the remaining methodological and interpretive concerns while preserving the study’s evidence-based contribution. We hope that the revised manuscript is now suitable for publication in PLOS ONE.

*Sincerely,*

**The Authors**
