# Supplementary material for: The hidden challenges of return migration: Linguistic and academic re-adaptation of Syrian students to the Arabic Education System
Source: PLoS One. 2026 Oct 16;21(10):e0361042. doi: 10.1371/journal.pone.0361042 (PMC13674759; doi:10.1371/journal.pone.0361042)
Supplement: Supplementary file 3 [file pone.0361042.s002.docx]

**RESPONSE TO REVIEWERS**

**Manuscript: The Hidden Challenges of Return Migration: Linguistic and Academic Reintegration of Syrian Returnee Students**

**Dear Editor and Reviewers of PLOS ONE,**

We are grateful to the editor and to all four reviewers for the time and care they devoted to our manuscript. The comments were constructive and thorough, and they have helped us improve the methodological transparency, theoretical grounding, and overall clarity of the study. We have revised the manuscript accordingly, and all changes are visible as tracked changes in the revised file. Below we respond to each comment in turn and indicate where the corresponding revision can be found. We hope that these revisions address the concerns raised and bring the manuscript closer to the standards of PLOS ONE.

**Response to the Editor**

**Data availability.** *All data underlying the findings should be made available, with rare exceptions such as participant privacy.*

**Response.** We added the data as support information in a word file.

**Recommended citations.** *Please evaluate any reviewer-recommended citations for relevance.*

**Response.** Reviewer 2 recommended fuller engagement with Cummins' framework, which we judged to be highly relevant and have incorporated into the literature review and Discussion. We also added recent literature in response to Reviewer 1. No other works were mandated for citation.

**ORCID.** *Co-authors are encouraged to verify their ORCID iDs before final acceptance.*

**Response.** Thank you for the reminder. We have asked all co-authors to verify their ORCID iDs within the submission system before final acceptance.

**Response to Reviewer 1**

We thank Reviewer 1 for the positive assessment and for the helpful suggestions for minor revision, each of which we have addressed.

***Comment:*** *Minor revisions are recommended to improve clarity in methodology, participant selection, and presentation of themes. Strengthening the discussion with additional recent literature and refining language consistency would further enhance the overall quality and readability of the manuscript.*

**Response.** We thank Reviewer 1 for the positive assessment and for these helpful suggestions, each of which we have addressed. To improve methodological clarity and participant selection, we expanded the Research Design and Participants sections with an explicit rationale for the phenomenological design, a clear statement of the inclusion criteria, and a justification for the participant age range. To strengthen the presentation of themes, we added a closing interpretive paragraph to each theme in the Findings that reads its broader educational and sociolinguistic significance, and the Discussion develops these readings further through the literature. To strengthen the discussion with recent literature, we incorporated four recent studies on the educational reintegration of returnee children from different national contexts (Maseko & Siziba, 2024 [46]; Grosa & King, 2023 [47]; Wang, 2025 [48]; Saghin et al., 2025 [49]). Finally, we revised the manuscript throughout to improve language consistency and readability.

**Response to Reviewer 2**

We thank Reviewer 2 for the detailed and rigorous review, which has substantially strengthened the methodological transparency and theoretical grounding of the paper.

**Comment 1 (Methodological concerns; participant age).** *Provide clearer justification for the analytical framework and explain how rigour was ensured with 15 participants; justify whether participants aged 11–14 can provide sufficiently rich data.*

**Response.** We expanded the Research Design section to clarify that the aim was not statistical generalization but an in-depth, context-sensitive understanding of students' lived experiences, and we clarified the relationship between phenomenology and content analysis. We strengthened the Trustworthiness section with additional detail on the audit trail, independent coding of 40% of the responses, member checking with four participants, and an intercoder agreement of 86% calculated using Miles and Huberman's formula. We also justified the participant age range, explaining that the school experiences, peer relations, and language difficulties under study are concrete, directly experienced phenomena that middle-school students can describe meaningfully, and that the questions were designed to be age-appropriate, concrete, and experience-based.

**Comment 2 (Data collection instrument).** *Critically discuss the limitations of written online responses, and include the full survey protocol with demographic information.*

**Response.** We added a discussion of the limitations of written online responses, including the absence of follow-up probing, both in the Data Collection section and in the new Limitations section. The complete seven-question protocol is retained in full, together with the participant demographic table, to support transparency and replicability.

**Comment 3 (Results and interpretation).** *The findings section requires deeper interpretation and analytical engagement. At present, the themes are presented descriptively without sufficient interpretation of their broader educational or sociolinguistic significance.*

**Response.** We thank the reviewer for this valuable observation. In response, we added a closing interpretive paragraph to each of the four themes. Rather than revising the descriptive presentation of individual extracts, we chose to add a synthesising paragraph at the end of each theme that draws its sub-themes together and reads them directly for their educational and sociolinguistic significance. We found this approach preferable because reworking the descriptive passages would have diluted the analytical tone, whereas a dedicated closing paragraph allowed us to articulate the deeper, more critical meaning of each theme clearly. We also believe that such summative interpretive paragraphs are well suited to qualitative research and improve the readability and coherence of the Findings section, and we are grateful to the reviewer for prompting this improvement.

**Comment 4 (Discussion)** *Moreover, references should generally not appear in the results section unless they are part of participants’ narratives or analytical framing. The results section should primarily focus on presenting and interpreting the study’s findings, while engagement with previous literature should be reserved for the discussion section.*

**Response.** Following this suggestion, we removed citations from the Findings section so that the results now focus on presenting and interpreting participants' data, while engagement with the literature is reserved for the Discussion. (This also aligns with our revision to the earlier Cummins reference; see Comment 6.) Building on the interpretive paragraphs now added at the end of each theme in the Findings, the Discussion develops these readings further by connecting them to the wider literature. The linguistic findings are read through the distinction between conversational fluency and academic language proficiency (Cummins); the social and pedagogical findings are connected to recent evidence that teachers in return contexts are often unprepared for re-entering students and that the relational climate of the classroom is central to post-return recovery (Grosa & King, 2023 [47]; Wang, 2025 [48]); and the psychosocial findings are interpreted as constitutive of the reverse academic transition rather than incidental to it (Saghin et al., 2025 [49]).

**Comment 5 (Conclusion overstates implications).** *The conclusion section overstates the implications of the study given the qualitative design and limited sample size. The authors should avoid presenting the findings as broadly representative and instead frame them as preliminary insights into the experiences of a specific group of participants. The language in the conclusion sections should therefore be moderated to better reflect the exploratory and context-specific nature of qualitative inquiry.*

**Response.** We moderated the Conclusion accordingly. Assertive verbs were softened (e.g., 'establishes' to 'suggests', 'constitutes' to 'may constitute'), the characterization of the reverse academic transition is now explicitly limited to the participants of this study, and we added a closing statement framing the conclusions as preliminary insights into a specific group rather than broadly generalizable claims. The recommendations are now presented as potential rather than essential pathways.

**Comment 6 (Cummins not introduced).** *In addition, the manuscript refers to the “linguistic disjunction” described by Cummins, yet Cummins is not sufficiently introduced or discussed in the literature review. Relevant theoretical foundations should be incorporated earlier in the paper.*

**Response.** Cummins' framework is now introduced in the literature review, where we explain the distinction between conversational fluency and academic language proficiency and the cross-linguistic interdependence principle. We also corrected the earlier Findings sentence that inaccurately attributed an 'asymmetric language development' model to Cummins; it has been rephrased descriptively, with the theoretical citation relocated to the literature review and Discussion, where it properly belongs.

**Comment 7 (Limitations and Future Research)** *Limitations and Future Research The manuscript does not sufficiently address limitations and directions for future research. A dedicated section discussing methodological limitations, sample size constraints, participant age considerations, and data collection limitations would strengthen the study. Suggestions for future research involving larger samples, triangulated data collection methods, or longitudinal designs would also be valuable.*

**Response.** We thank the reviewer for this helpful suggestion. We have added a dedicated "Limitations and Future Research" section, placed between the Discussion and the Conclusion. This section explicitly addresses the methodological limitations of the study, including the relatively small and context-specific sample, the age range of the participants and its implications for the depth of reflection, and the constraints of collecting data through written online responses rather than face-to-face interviews. It also outlines directions for future research, including the use of larger and more diverse samples, triangulated data-collection methods such as interviews, classroom observations, and teacher and parent perspectives, and longitudinal designs that follow students over time. We are grateful to the reviewer for prompting this addition, which we believe strengthens the study.

**Response to Reviewer 3**

We are grateful to Reviewer 3 for the encouraging assessment and for the clear, practical suggestions.

**Comment 1 (Face validity).** *There should be more on the face validity of the survey. There is not much information about that.*

**Response.** We added information on face validity to the Data Collection section. In addition to the expert review conducted for content validity, the pilot administration established the face validity of the protocol: the two pilot students evaluated whether the questions were clear, comprehensible, and meaningful from the perspective of the target age group, and their feedback informed minor wording revisions.

**Comment 2 (Incentives).** *The manuscript should mention whether the participants received any incentives or not.*

**Response.** No incentives or compensation of any kind were provided to participants; this is now stated explicitly.

**Comment 3 (Time to complete).** *The manuscript should mention how much time did it took to fill out the survey.***Response.** Completing the survey took participants approximately 15 to 25 minutes; this is now reported in the Data Collection section.

**Comment 4 (Code table and data saturation).** *There should be a table that shows the codes and subcodes and who mentioned what from the participants and how did that progressed over the interviews to see if the research team was able to achieve data saturation.*

**Response.** We thank the reviewer for this valuable suggestion. We have added two tables to address it. Table 2 presents the twelve sub-codes together with the participants whose accounts contributed to each, making transparent which participants are reflected in each sub-code. Table 3 then shows how coding progressed across participants: all twelve sub-codes had emerged by the fifth participant, and no new sub-codes were identified thereafter, indicating that data saturation was reached well before the end of the data set. We believe these additions directly address the reviewer's request and strengthen the transparency and trustworthiness of the analysis.

**Response to Reviewer 4**

We thank Reviewer 4 for the constructive suggestions, which have improved the structure and formal quality of the manuscript.

**Comment 1 (Editing).** *Editing is highly recommended.*

**Response.** The manuscript has been carefully edited for grammar, word choice, and consistency, and we revised a number of sentences to improve academic readability while preserving the original meaning.

**Comment 2 (Research questions and abstract).** *The research questions should be introduced in the abstract and at the end of the Introduction section. Besides, the abstract should contain the research gap, significance, research questions/objectives, model/instruments used, findings, and some pedagogical imlpications.*

**Response.** The overarching research question and four sub-questions are now stated explicitly at the end of the Introduction. The abstract was revised to include the research gap, the research question, the instrument (a seven-item open-ended written survey), the phenomenological design, the main findings, and the pedagogical implications.

**Comment 3 ('Figure' vs 'Fig').** *Using a full form such as Figure is more formal than the abbreviated form. Apply to all such cases.*

**Response.** We appreciate this point. PLOS ONE's style guidelines, however, require the abbreviated form 'Fig' for in-text figure citations (e.g., 'Fig 1'), reserving the full form only at the beginning of a sentence. To remain compliant with the journal's house style we have retained 'Fig' and applied it consistently throughout; we are of course happy to adjust if the editor prefers otherwise.

**Comment 4 (Separate Conclusion).** *The conclusion should be a separate section.*

**Response.** The previously combined 'Discussion and conclusion' section has been divided into separate 'Discussion' and 'Conclusion' sections, with a new 'Limitations and Future Research' section placed between them.

**Comment 5 (Limitations).** *A section on Limitations and suggestions can be added.*

**Response.** A 'Limitations and Future Research' section has been added. Thank you very much for this recommendation.

We thank the editor and the reviewers once again for their valuable feedback, which has clearly strengthened the manuscript. We hope that the revised version is now suitable for publication in PLOS ONE and look forward to your decision.

Sincerely,

**The Authors**
