# Supplementary material for: The hidden challenges of return migration: Linguistic and academic re-adaptation of Syrian students to the Arabic Education System
Source: PLoS One. 2026 Oct 16;21(10):e0361042. doi: 10.1371/journal.pone.0361042 (PMC13674759; doi:10.1371/journal.pone.0361042)
Supplement: Peer review history

# Peer review: Decision Letter 0

**DOI:** 10.1371/journal.pone.0361042.r001

**Authors:** Ömer Gökhan Ulum

8 Jun 2026

PONE-D-26-11047

The Hidden Challenges of Return Migration: Linguistic and Academic Re-Adaptation of Syrian Students to the Arabic Education System

PLOS One

Dear Dr. Dolapcioglu,

Thank you for submitting your manuscript to PLOS ONE. After careful consideration, we feel that it has merit but does not fully meet PLOS ONE’s publication criteria as it currently stands. Therefore, we invite you to submit a revised version of the manuscript that addresses the points raised during the review process.

==============================

Dear Author,

Thank you for submitting your manuscript.

After careful evaluation, the reviewers have provided comments and suggestions that should be addressed before the manuscript can be considered for further processing. Please revise your paper in accordance with the reviewers’ feedback and prepare a detailed point-by-point response explaining how each comment has been addressed in the revised version.

Kindly submit both the revised manuscript and the response-to-reviewers document by the specified deadline.

We appreciate your efforts in improving the quality of your work and look forward to receiving your revised submission.

==============================

Please submit your revised manuscript by Jul 17 2026 11:59PM. If you will need more time than this to complete your revisions, please reply to this message or contact the journal office at plosone@plos.org. When you're ready to submit your revision, log on to https://www.editorialmanager.com/pone/ and select the 'Submissions Needing Revision' folder to locate your manuscript file.

Please include the following items when submitting your revised manuscript:

- A letter that responds to each point raised by the academic editor and reviewer(s). You should upload this letter as a separate file labeled 'Response to Reviewers'.
- A marked-up copy of your manuscript that highlights changes made to the original version. You should upload this as a separate file labeled 'Revised Manuscript with Track Changes'.
- An unmarked version of your revised paper without tracked changes. You should upload this as a separate file labeled 'Manuscript'.

If you would like to make changes to your financial disclosure, please include your updated statement in your cover letter. Guidelines for resubmitting your figure files are available below the reviewer comments at the end of this letter.

If applicable, we recommend that you deposit your laboratory protocols in protocols.io to enhance the reproducibility of your results. Protocols.io assigns your protocol its own identifier (DOI) so that it can be cited independently in the future. For instructions see: https://journals.plos.org/plosone/s/submission-guidelines#loc-laboratory-protocols. Additionally, PLOS ONE offers an option for publishing peer-reviewed Lab Protocol articles, which describe protocols hosted on protocols.io. Read more information on sharing protocols at https://plos.org/protocols?utm_medium=editorial-email&utm_source=authorletters&utm_campaign=protocols.

As the corresponding author, your ORCID iD is verified in the submission system and will appear in the published article. PLOS supports the use of ORCID, and we encourage all coauthors to register for an ORCID iD and use it as well. Please encourage your coauthors to verify their ORCID iD within the submission system before final acceptance, as unverified ORCID iDs will not appear in the published article. Only the individual author can complete the verification step; PLOS staff cannot verify ORCID iDs on behalf of authors.

We look forward to receiving your revised manuscript.

Kind regards,

Ömer Gökhan Ulum

Academic Editor

PLOS One

Journal Requirements:

When submitting your revision, we need you to address these additional requirements.

1. Please ensure that your manuscript meets PLOS ONE's style requirements, including those for file naming. The PLOS ONE style templates can be found at

https://journals.plos.org/plosone/s/file?id=wjVg/PLOSOne_formatting_sample_main_body.pdf and

https://journals.plos.org/plosone/s/file?id=ba62/PLOSOne_formatting_sample_title_authors_affiliations.pdf

2. We note that you have indicated that there are restrictions to data sharing for this study. PLOS only allows data to be available upon request if there are legal or ethical restrictions on sharing data publicly. For more information on unacceptable data access restrictions, please see http://journals.plos.org/plosone/s/data-availability#loc-unacceptable-data-access-restrictions.

Before we proceed with your manuscript, please address the following prompts:

a) If there are ethical or legal restrictions on sharing a de-identified data set, please explain them in detail (e.g., data contain potentially identifying or sensitive patient information, data are owned by a third-party organization, etc.) and who has imposed them (e.g., a Research Ethics Committee or Institutional Review Board, etc.). Please also provide contact information for a data access committee, ethics committee, or other institutional body to which data requests may be sent.

b) If there are no restrictions, please upload the minimal anonymized data set necessary to replicate your study findings to a stable, public repository and provide us with the relevant URLs, DOIs, or accession numbers. For a list of recommended repositories, please see

https://journals.plos.org/plosone/s/recommended-repositories. You also have the option of uploading the data as Supporting Information files, but we would recommend depositing data directly to a data repository if possible.

We will update your Data Availability statement on your behalf to reflect the information you provide.

3. Please include captions for your Supporting Information files at the end of your manuscript, and update any in-text citations to match accordingly. Please see our Supporting Information guidelines for more information: http://journals.plos.org/plosone/s/supporting-information.

4. If the reviewer comments include a recommendation to cite specific previously published works, please review and evaluate these publications to determine whether they are relevant and should be cited. There is no requirement to cite these works unless the editor has indicated otherwise.

[Note: HTML markup is below. Please do not edit.]

Reviewers' comments:

Reviewer's Responses to Questions

**Comments to the Author**

1. Is the manuscript technically sound, and do the data support the conclusions?

Reviewer #1: Yes

Reviewer #2: Partly

Reviewer #3: Yes

Reviewer #4: Yes

**********

2. Has the statistical analysis been performed appropriately and rigorously?

Reviewer #1: Yes

Reviewer #2: N/A

Reviewer #3: N/A

Reviewer #4: I Don't Know

**********

3. Have the authors made all data underlying the findings in their manuscript fully available?

The PLOS Data policy

Reviewer #1: Yes

Reviewer #2: Yes

Reviewer #3: Yes

Reviewer #4: No

**********

4. Is the manuscript presented in an intelligible fashion and written in standard English?

Reviewer #1: Yes

Reviewer #2: Yes

Reviewer #3: Yes

Reviewer #4: Yes

**********

Reviewer #1: The manuscript addresses an important and underexplored dimension of refugee education by focusing on post-return educational reintegration. The qualitative findings are meaningful and provide valuable insights into linguistic, academic, and psychosocial challenges faced by Syrian returnee students. The study is well-structured and theoretically relevant. However, minor revisions are recommended to improve clarity in methodology, participant selection, and presentation of themes. Strengthening the discussion with additional recent literature and refining language consistency would further enhance the overall quality and readability of the manuscript.

Reviewer #2: Review Report

Manuscript Title: The Hidden Challenges of Return Migration: Linguistic and Academic Re-Adaptation of Syrian Students to the Arabic Education System

The manuscript addresses an important and timely topic related to the linguistic and academic re-adaptation of Syrian returnee students within the Arabic education system. The study also tackles an underexplored area in migration and educational research, and the literature review is generally solid, with a clearly identified research gap that the study attempts to fill. Nevertheless, several methodological and interpretive concerns need to be addressed before the paper can be considered for publication.

Major Comments

1. Methodological Concerns

The study adopts a phenomenological approach, which is appropriate for exploring lived experiences. However, the use of content analysis with only 15 participants raises methodological concerns. The manuscript should provide a clearer justification for the chosen analytical framework and explain how rigorous qualitative analysis was ensured with such a limited sample.

Additionally, the participants are middle-school students aged between 11 and 14 years. The authors should justify whether participants at this age can provide sufficiently rich, reflective, and nuanced qualitative data required for phenomenological inquiry.

2. Data Collection Instrument

The manuscript states that a Google Forms survey with open-ended questions was used as the primary data collection instrument because face-to-face interviews were not feasible. While this adaptation may be understandable, the limitations of relying solely on written online responses from young participants should be critically discussed.

Furthermore, the final protocol reportedly consisted of seven open-ended questions aligned with the research questions; however, these questions are not included in the manuscript. The inclusion of the full interview/survey protocol is necessary for transparency and replicability with the section about demographics.

3. Results and Interpretation

The findings section requires deeper interpretation and analytical engagement. At present, the themes are presented descriptively without sufficient interpretation of their broader educational or sociolinguistic significance.

In addition, the manuscript refers to the “linguistic disjunction” described by Cummins, yet Cummins is not sufficiently introduced or discussed in the literature review. Relevant theoretical foundations should be incorporated earlier in the paper.

Moreover, references should generally not appear in the results section unless they are part of participants’ narratives or analytical framing. The results section should primarily focus on presenting and interpreting the study’s findings, while engagement with previous literature should be reserved for the discussion section.

4. Conclusion and Implications

The conclusion section overstates the implications of the study given the qualitative design and limited sample size. The authors should avoid presenting the findings as broadly representative and instead frame them as preliminary insights into the experiences of a specific group of participants. The language in the conclusion sections should therefore be moderated to better reflect the exploratory and context-specific nature of qualitative inquiry.

7. Limitations and Future Research

The manuscript does not sufficiently address limitations and directions for future research. A dedicated section discussing methodological limitations, sample size constraints, participant age considerations, and data collection limitations would strengthen the study. Suggestions for future research involving larger samples, triangulated data collection methods, or longitudinal designs would also be valuable.

Strengths of the Manuscript

The study addresses an important and underexplored topic.

The literature review is well-developed and identifies a meaningful research gap.

The paper has potential to contribute to discussions on migration, education, and linguistic adaptation if the methodological and interpretive concerns are adequately addressed.

Recommendation

Major revisions required.

Reviewer #3: This is a very well-written paper. Congratulations to the authors!

I have a few comments and easy fixes:

1. There should be more on the face validity of the survey. There is not much information about that.

2. The manuscript should mention whether the participants received any incentives or not.

3. The manuscript should mention how much time did it took to fill out the survey.

4. There should be a table that shows the codes and subcodes and who mentioned what from the participants and how did that progressed over the interviews to see if the research team was able to achieve data saturation.

Reviewer #4: Dear author,

The topic is interesting and tackling a sensitive issue, yet the following are to reconsider:

1.Editing is highly recommended.

2.The research questions should be introduced in the abstract and at the end of the Introduction section. Besides, the abstract should contain the research gap, significance, research questions/objectives, model/instruments used, findings, and some pedagogical imlpications.

3.Using a full form such as Figure is more formal than the abbreviated form. Apply to all such cases.

4.The conclusion should be a separate section.

5.A section on Limitations and suggestions can be added.

6.Rgs.

**********

what does this mean?). If published, this will include your full peer review and any attached files.

If you choose “no”, your identity will remain anonymous but your review may still be made public.

**Do you want your identity to be public for this peer review?**  For information about this choice, including consent withdrawal, please see our Privacy Policy

Reviewer #1: No

Reviewer #2: No

Reviewer #3: **Yes:**  Amer Hamad Issa Abukhalaf

Reviewer #4: **Yes:**  Nawal Fadhil Abbas

**********

[NOTE: If reviewer comments were submitted as an attachment file, they will be attached to this email and accessible via the submission site. Please log into your account, locate the manuscript record, and check for the action link "View Attachments". If this link does not appear, there are no attachment files.]

To ensure your figures meet our technical requirements, please review our figure guidelines: https://journals.plos.org/plosone/s/figures

You may also use PLOS’s free figure tool, NAAS, to help you prepare publication quality figures: https://journals.plos.org/plosone/s/figures#loc-tools-for-figure-preparation.

NAAS will assess whether your figures meet our technical requirements by comparing each figure against our figure specifications.

# Author comment: Author response to Decision Letter 1

**DOI:** 10.1371/journal.pone.0361042.r002

27 Jul 2026

Response to Reviewers

Manuscript: The Hidden Challenges of Return Migration: Linguistic and Academic Reintegration of Syrian Returnee Students

Dear Editor and Reviewers of PLOS ONE,

We are grateful to the editor and to all four reviewers for the time and care they devoted to our manuscript. The comments were constructive and thorough, and they have helped us improve the methodological transparency, theoretical grounding, and overall clarity of the study. We have revised the manuscript accordingly, and all changes are visible as tracked changes in the revised file. Below we respond to each comment in turn and indicate where the corresponding revision can be found. We hope that these revisions address the concerns raised and bring the manuscript closer to the standards of PLOS ONE.

Response to the Editor

Data availability. All data underlying the findings should be made available, with rare exceptions such as participant privacy.

Response. BURASI SENDE. HAM VERİYİ YÜKLEMEK GEREKİYOR CEVABINI DA BURAYA SUPPLEMENTARY FILE OLARAK YA DA NASIL OLDUYSA ÖYLE YAZMAK GEREKİR EMAIL DE TARIF EDİYORDU SANKİ.

Recommended citations. Please evaluate any reviewer-recommended citations for relevance.

Response. Reviewer 2 recommended fuller engagement with Cummins' framework, which we judged to be highly relevant and have incorporated into the literature review and Discussion. We also added recent literature in response to Reviewer 1. No other works were mandated for citation.

ORCID. Co-authors are encouraged to verify their ORCID iDs before final acceptance.

Response. Thank you for the reminder. We have asked all co-authors to verify their ORCID iDs within the submission system before final acceptance.

Response to Reviewer 1

We thank Reviewer 1 for the positive assessment and for the helpful suggestions for minor revision, each of which we have addressed.

Comment: Minor revisions are recommended to improve clarity in methodology, participant selection, and presentation of themes. Strengthening the discussion with additional recent literature and refining language consistency would further enhance the overall quality and readability of the manuscript.

Response. We thank Reviewer 1 for the positive assessment and for these helpful suggestions, each of which we have addressed. To improve methodological clarity and participant selection, we expanded the Research Design and Participants sections with an explicit rationale for the phenomenological design, a clear statement of the inclusion criteria, and a justification for the participant age range. To strengthen the presentation of themes, we added a closing interpretive paragraph to each theme in the Findings that reads its broader educational and sociolinguistic significance, and the Discussion develops these readings further through the literature. To strengthen the discussion with recent literature, we incorporated four recent studies on the educational reintegration of returnee children from different national contexts (Maseko & Siziba, 2024 [46]; Grosa & King, 2023 [47]; Wang, 2025 [48]; Saghin et al., 2025 [49]). Finally, we revised the manuscript throughout to improve language consistency and readability.

Response to Reviewer 2

We thank Reviewer 2 for the detailed and rigorous review, which has substantially strengthened the methodological transparency and theoretical grounding of the paper.

Comment 1 (Methodological concerns; participant age). Provide clearer justification for the analytical framework and explain how rigour was ensured with 15 participants; justify whether participants aged 11–14 can provide sufficiently rich data.

Response. We expanded the Research Design section to clarify that the aim was not statistical generalization but an in-depth, context-sensitive understanding of students' lived experiences, and we clarified the relationship between phenomenology and content analysis. We strengthened the Trustworthiness section with additional detail on the audit trail, independent coding of 40% of the responses, member checking with four participants, and an intercoder agreement of 86% calculated using Miles and Huberman's formula. We also justified the participant age range, explaining that the school experiences, peer relations, and language difficulties under study are concrete, directly experienced phenomena that middle-school students can describe meaningfully, and that the questions were designed to be age-appropriate, concrete, and experience-based.

Comment 2 (Data collection instrument). Critically discuss the limitations of written online responses, and include the full survey protocol with demographic information.

Response. We added a discussion of the limitations of written online responses, including the absence of follow-up probing, both in the Data Collection section and in the new Limitations section. The complete seven-question protocol is retained in full, together with the participant demographic table, to support transparency and replicability.

Comment 3 (Results and interpretation). The findings section requires deeper interpretation and analytical engagement. At present, the themes are presented descriptively without sufficient interpretation of their broader educational or sociolinguistic significance.

Response. We thank the reviewer for this valuable observation. In response, we added a closing interpretive paragraph to each of the four themes. Rather than revising the descriptive presentation of individual extracts, we chose to add a synthesising paragraph at the end of each theme that draws its sub-themes together and reads them directly for their educational and sociolinguistic significance. We found this approach preferable because reworking the descriptive passages would have diluted the analytical tone, whereas a dedicated closing paragraph allowed us to articulate the deeper, more critical meaning of each theme clearly. We also believe that such summative interpretive paragraphs are well suited to qualitative research and improve the readability and coherence of the Findings section, and we are grateful to the reviewer for prompting this improvement.

Comment 4 (Discussion) Moreover, references should generally not appear in the results section unless they are part of participants’ narratives or analytical framing. The results section should primarily focus on presenting and interpreting the study’s findings, while engagement with previous literature should be reserved for the discussion section.

Response. Following this suggestion, we removed citations from the Findings section so that the results now focus on presenting and interpreting participants' data, while engagement with the literature is reserved for the Discussion. (This also aligns with our revision to the earlier Cummins reference; see Comment 6.) Building on the interpretive paragraphs now added at the end of each theme in the Findings, the Discussion develops these readings further by connecting them to the wider literature. The linguistic findings are read through the distinction between conversational fluency and academic language proficiency (Cummins); the social and pedagogical findings are connected to recent evidence that teachers in return contexts are often unprepared for re-entering students and that the relational climate of the classroom is central to post-return recovery (Grosa & King, 2023 [47]; Wang, 2025 [48]); and the psychosocial findings are interpreted as constitutive of the reverse academic transition rather than incidental to it (Saghin et al., 2025 [49]).

Comment 5 (Conclusion overstates implications). The conclusion section overstates the implications of the study given the qualitative design and limited sample size. The authors should avoid presenting the findings as broadly representative and instead frame them as preliminary insights into the experiences of a specific group of participants. The language in the conclusion sections should therefore be moderated to better reflect the exploratory and context-specific nature of qualitative inquiry.

Response. We moderated the Conclusion accordingly. Assertive verbs were softened (e.g., 'establishes' to 'suggests', 'constitutes' to 'may constitute'), the characterization of the reverse academic transition is now explicitly limited to the participants of this study, and we added a closing statement framing the conclusions as preliminary insights into a specific group rather than broadly generalizable claims. The recommendations are now presented as potential rather than essential pathways.

Comment 6 (Cummins not introduced). In addition, the manuscript refers to the “linguistic disjunction” described by Cummins, yet Cummins is not sufficiently introduced or discussed in the literature review. Relevant theoretical foundations should be incorporated earlier in the paper.

Response. Cummins' framework is now introduced in the literature review, where we explain the distinction between conversational fluency and academic language proficiency and the cross-linguistic interdependence principle. We also corrected the earlier Findings sentence that inaccurately attributed an 'asymmetric language development' model to Cummins; it has been rephrased descriptively, with the theoretical citation relocated to the literature review and Discussion, where it properly belongs.

Comment 7 (Limitations and Future Research) Limitations and Future Research

The manuscript does not sufficiently address limitations and directions for future research. A dedicated section discussing methodological limitations, sample size constraints, participant age considerations, and data collection limitations would strengthen the study. Suggestions for future research involving larger samples, triangulated data collection methods, or longitudinal designs would also be valuable.

Response. We thank the reviewer for this helpful suggestion. We have added a dedicated "Limitations and Future Research" section, placed between the Discussion and the Conclusion. This section explicitly addresses the methodological limitations of the study, including the relatively small and context-specific sample, the age range of the participants and its implications for the depth of reflection, and the constraints of collecting data through written online responses rather than face-to-face interviews. It also outlines directions for future research, including the use of larger and more diverse samples, triangulated data-collection methods such as interviews, classroom observations, and teacher and parent perspectives, and longitudinal designs that follow students over time. We are grateful to the reviewer for prompting this addition, which we believe strengthens the study.

Response to Reviewer 3

We are grateful to Reviewer 3 for the encouraging assessment and for the clear, practical suggestions.

Comment 1 (Face validity). There should be more on the face validity of the survey. There is not much information about that.

Response. We added information on face validity to the Data Collection section. In addition to the expert review conducted for content validity, the pilot administration established the face validity of the protocol: the two pilot students evaluated whether the questions were clear, comprehensible, and meaningful from the perspective of the target age group, and their feedback informed minor wording revisions.

Comment 2 (Incentives). The manuscript should mention whether the participants received any incentives or not.

Response. No incentives or compensation of any kind were provided to participants; this is now stated explicitly.

Comment 3 (Time to complete). The manuscript should mention how much time did it took to fill out the survey.

Response. Completing the survey took participants approximately 15 to 25 minutes; this is now reported in the Data Collection section.

Comment 4 (Code table and data saturation). There should be a table that shows the codes and subcodes and who mentioned what from the participants and how did that progressed over the interviews to see if the research team was able to achieve data saturation.

Response. We thank the reviewer for this valuable suggestion. We have added two tables to address it. Table 2 presents the twelve sub-codes together with the participants whose accounts contributed to each, making transparent which participants are reflected in each sub-code. Table 3 then shows how coding progressed across participants: all twelve sub-codes had emerged by the fifth participant, and no new sub-codes were identified thereafter, indicating that data saturation was reached well before the end of the data set. We believe these additions directly address the reviewer's request and strengthen the transparency and trustworthiness of the analysis. BEN BÖYLE BİR CEVAP HAZIRLADIM AMA BUNLAR TABLO OLARAK MI KALSIN, APPENDIX MI KARAR VEREMİYORUM. SANA BIRAKIYORUM. TABLO DERSEN METİN İÇİNDEKİ TABLO NUMARALARINI UNUTMA LÜTFEN. AYRICA, BU TABLOLARI KONTROL ETMENDE FAYDA VAR. BEN BENDEKİ BULGU VE KODLAR İLE İLERLEDİM. ZATEN S14 VE S15DE İŞARET OLMADIĞINI GÖRECEKSİN, ONLARA DA İŞARET KOYMAK GEREKEBİLİR, ELİNDEKİ VERİYE GÖRE HAREKET EDECEĞİN İÇİN SANA BIRAKIYORUM.

Response to Reviewer 4

We thank Reviewer 4 for the constructive suggestions, which have improved the structure and formal quality of the manuscript.

Comment 1 (Editing). Editing is highly recommended.

Response. The manuscript has been carefully edited for grammar, word choice, and consistency, and we revised a number of sentences to improve academic readability while preserving the original meaning.

Comment 2 (Research questions and abstract). The research questions should be introduced in the abstract and at the end of the Introduction section. Besides, the abstract should contain the research gap, significance, research questions/objectives, model/instruments used, findings, and some pedagogical imlpications.

Response. The overarching research question and four sub-questions are now stated explicitly at the end of the Introduction. The abstract was revised to include the research gap, the research question, the instrument (a seven-item open-ended written survey), the phenomenological design, the main findings, and the pedagogical implications.

Comment 3 ('Figure' vs 'Fig'). Using a full form such as Figure is more formal than the abbreviated form. Apply to all such cases.

Response. We appreciate this point. PLOS ONE's style guidelines, however, require the abbreviated form 'Fig' for in-text figure citations (e.g., 'Fig 1'), reserving the full form only at the beginning of a sentence. To remain compliant with the journal's house style we have retained 'Fig' and applied it consistently throughout; we are of course happy to adjust if the editor prefers otherwise.

Comment 4 (Separate Conclusion). The conclusion should be a separate section.

Response. The previously combined 'Discussion and conclusion' section has been divided into separate 'Discussion' and 'Conclusion' sections, with a new 'Limitations and Future Research' section placed between them.

Comment 5 (Limitations). A section on Limitations and suggestions can be added.

Response. A 'Limitations and Future Research' section has been added. Thank you very much for this recommendation.

We thank the editor and the reviewers once again for their valuable feedback, which has clearly strengthened the manuscript. We hope that the revised version is now suitable for publication in PLOS ONE and look forward to your decision.

Sincerely,

The Authors

Attachment Submitted filename: Response_to_Reviewers (2).docx

# Peer review: Decision Letter 1

**DOI:** 10.1371/journal.pone.0361042.r003

**Authors:** Ömer Gökhan Ulum

31 Aug 2026

Dear Dr.  Dolapcioglu,

Thank you for submitting your manuscript to PLOS One. After careful consideration, we feel that it has merit but does not fully meet PLOS One’s publication criteria as it currently stands. Therefore, we invite you to submit a revised version of the manuscript that addresses the points raised during the review process.

Please submit your revised manuscript by Oct 15 2026 11:59PM. If you will need more time than this to complete your revisions, please reply to this message or contact the journal office at plosone@plos.org. When you're ready to submit your revision, log on to https://www.editorialmanager.com/pone/ and select the 'Submissions Needing Revision' folder to locate your manuscript file.

- A letter that responds to each point raised by the academic editor and reviewer(s). You should upload this letter as a separate file labeled 'Response to Reviewers'.
- A marked-up copy of your manuscript that highlights changes made to the original version. You should upload this as a separate file labeled 'Revised Manuscript with Track Changes'.
- An unmarked version of your revised paper without tracked changes. You should upload this as a separate file labeled 'Manuscript'.

If you would like to make changes to your financial disclosure, please include your updated statement in your cover letter. Guidelines for resubmitting your figure files are available below the reviewer comments at the end of this letter.

If applicable, we recommend that you deposit your laboratory protocols in protocols.io to enhance the reproducibility of your results. Protocols.io assigns your protocol its own identifier (DOI) so that it can be cited independently in the future. For instructions see: https://journals.plos.org/plosone/s/submission-guidelines#loc-laboratory-protocols. Additionally, PLOS One offers an option for publishing peer-reviewed Lab Protocol articles, which describe protocols hosted on protocols.io. Read more information on sharing protocols at https://plos.org/protocols?utm_medium=editorial-email&utm_source=authorletters&utm_campaign=protocols.

As the corresponding author, your ORCID iD is verified in the submission system and will appear in the published article. PLOS supports the use of ORCID, and we encourage all coauthors to register for an ORCID iD and use it as well. Please encourage your coauthors to verify their ORCID iD within the submission system before final acceptance, as unverified ORCID iDs will not appear in the published article. *Only*  the individual author can complete the verification step; PLOS staff *cannot*  verify ORCID iDs on behalf of authors.

We look forward to receiving your revised manuscript.

Kind regards,

Ömer Gökhan Ulum

Academic Editor

PLOS One

Journal Requirements:

If the reviewer comments include a recommendation to cite specific previously published works, please review and evaluate these publications to determine whether they are relevant and should be cited. There is no requirement to cite these works unless the editor has indicated otherwise.

Please review your reference list to ensure that it is complete and correct. If you have cited papers that have been retracted, please include the rationale for doing so in the manuscript text, or remove these references and replace them with relevant current references. Any changes to the reference list should be mentioned in the rebuttal letter that accompanies your revised manuscript. If you need to cite a retracted article, indicate the article’s retracted status in the References list and also include a citation and full reference for the retraction notice.

Additional Editor Comments:

Decision: Minor Revision

Both reviewers consider the manuscript interesting, relevant, and methodologically sound, and they recognize its potential contribution to the literature on refugee education and the educational consequences of return migration. Overall, the manuscript has been substantially improved.

However, both reviewers have identified several minor issues that should be addressed before the manuscript can be accepted. In particular, the authors are requested to:

Provide a clearer justification for the phenomenological design and explain how phenomenological principles informed the data collection and analysis.

Revise potentially diagnostic or overly strong psychological terminology and adopt a more contextual and relational interpretation of students’ experiences.

Qualify broad statements about Turkish and Syrian educational systems so that interpretations remain grounded in the participants’ accounts.

Clarify the procedures and justification concerning data saturation, coding, and the development of the coding framework, and reconcile the relevant tables and supplementary materials.

Carefully proofread the manuscript for grammatical and typographical errors, ensure consistent terminology, and strengthen the discussion by engaging with a few relevant recent studies, including those suggested by the reviewers.

Please revise the manuscript carefully in accordance with the comments and recommendations of both reviewers.

[Note: HTML markup is below. Please do not edit.]

Reviewers' comments:

Reviewer's Responses to Questions

**Comments to the Author**

Reviewer #1: (No Response)

Reviewer #3: All comments have been addressed

Reviewer #5: (No Response)

**********

2. Is the manuscript technically sound, and do the data support the conclusions?

Reviewer #1: Yes

Reviewer #3: Yes

Reviewer #5: (No Response)

**********

3. Has the statistical analysis been performed appropriately and rigorously?

Reviewer #1: Yes

Reviewer #3: N/A

Reviewer #5: (No Response)

**********

4. Have the authors made all data underlying the findings in their manuscript fully available?

The PLOS Data policy

Reviewer #1: Yes

Reviewer #3: Yes

Reviewer #5: (No Response)

**********

5. Is the manuscript presented in an intelligible fashion and written in standard English?

Reviewer #1: Yes

Reviewer #3: Yes

Reviewer #5: (No Response)

**********

Reviewer #1: The manuscript addresses an important and underexplored topic concerning the educational reintegration of voluntarily returned Syrian students. The study is well designed, the methodology is appropriate, and the findings provide meaningful theoretical and practical contributions to refugee education and bilingual learning research. The discussion is coherent and the implications are clearly articulated. However, a few minor revisions are recommended before acceptance. The authors should carefully proofread the manuscript for minor grammatical and typographical errors, ensure consistent terminology throughout the text, and strengthen the discussion by explicitly linking the findings to a few additional recent studies. Subject to these minor corrections, the manuscript is suitable for publication.

Reviewer #3: I have no further Comments. All my comments have been addressed and I am happy with the end results of the paper.

Reviewer #5: This is an interesting paper that shifts attention from refugee integration in host countries to the comparatively neglected educational consequences of return migration, particularly the transition from Turkish-medium to Arabic-medium schooling. The revision has clearly improved the paper, especially in its articulation of the research questions, acknowledgement of limitations, expansion of the theoretical discussion, and description of trustworthiness procedures. However, I still see some issues that should be addressed before publication. I have specifies these issues bellow:

1. The manuscript identifies the study as phenomenological, yet the empirical material was generated through seven open-ended questions administered through Google Forms to 15 students aged 11–14. Although the authors appropriately explain that geographical constraints prevented face-to-face interviews, written responses collected within approximately 15–25 minutes necessarily provide limited opportunities for probing, clarification, and deeper exploration of lived experience. The authors should therefore provide a more convincing explanation of what specifically makes the study phenomenological rather than simply an exploratory qualitative study employing qualitative content analysis. This should include identification of the particular phenomenological orientation adopted and a clearer explanation of how phenomenological principles informed both data collection and interpretation. The authors may find it useful to engage with empirical examples demonstrating how phenomenological inquiry can connect participants’ lived experiences, contextual conditions, and meaning-making processes while maintaining methodological coherence; for example, Soomro et al. (2025) may provide a relevant methodological point of comparison in the field of ESL and education.

2. The manuscript sometimes uses terminology that approaches psychological diagnosis despite the absence of a trauma-specific instrument, clinical assessment, or in-depth psychological interviews. Statements describing concentration difficulties as a “classic effect of trauma,” raised voices as trauma “triggers,” or students as having weakened psychological resilience go beyond what can confidently be inferred from short written accounts. The authors should distinguish more carefully between what participants actually reported and the researchers’ interpretation through the trauma literature. Terms such as “migration-related distress,” “psychosocial strain,” or “experiences potentially consistent with trauma-related responses” would be more defensible. More broadly, the paper would benefit from adopting a relational and contextual interpretation of students’ experiences rather than treating their responses primarily as individual psychological conditions. In this respect, the authors may consider the structure–agency perspective developed by Rind (2016), which emphasises how learners’ experiences emerge through interactions among individual agency, institutional structures, social relationships, and contextual constraints. Such a perspective could complement the manuscript’s existing argument that returnees’ difficulties are produced partly through institutional mismatches rather than individual deficits.

3. Several sections move from students’ individual accounts to rather broad characterisations of Turkish schooling as student-centred and interactive and Syrian schooling as teacher-centred, traditional, transmission-oriented, or authoritarian. For instance, students’ experiences of copying from the board, limited experimentation, and rapid instructional pacing are interpreted as evidence of a broader transition between contrasting pedagogical systems. The data support claims about how these particular students perceived differences between their previous and current classrooms, but a sample of 15 students cannot support general statements about two national education systems. The authors should therefore consistently qualify these interpretations—for example, “participants perceived their current classrooms as more teacher-centred than the classrooms they had previously experienced in Türkiye.” Similar caution is required when discussing curriculum quality, assessment transparency, teacher authority, and pedagogical culture.

4. The response to reviewers states that all twelve sub-codes had emerged by the fifth participant and that no new codes appeared subsequently, which is interpreted as evidence that saturation had been achieved. This claim requires further methodological justification. The authors should explain the order in which responses were collected and analysed, whether coding occurred concurrently with recruitment, how saturation was operationally defined, and whether the sample size was predetermined. In a fixed dataset analysed retrospectively, observing that later participants generated no new codes is not necessarily equivalent to demonstrating prospective theoretical or meaning saturation. Moreover, there appears to be inconsistency between the response letter—which indicates that dedicated participant/code and saturation tables were added—and the clean manuscript, where Tables 2–5 represent the four substantive themes. The authors should reconcile the table numbering and provide the coding/saturation material as supplementary information if necessary. Importantly, all participant-by-code entries must derive directly from the underlying data rather than being retrospectively completed to create a more symmetrical coding matrix.

Rind, I. A. (2016). Conceptualizing students’ learning experiences in English as a second language in higher education from structure and agency. Cogent Social Sciences, 2, 1191978.

Soomro, R. B. K., Khan, R. A., & Rind, I. A. (2025). Exploring ESL teachers’ experiences about ICT-based 4Cs skills: A phenomenological study. Discover Education, 4(12).

**********

what does this mean?). If published, this will include your full peer review and any attached files.

If you choose “no”, your identity will remain anonymous but your review may still be made public.

**Do you want your identity to be public for this peer review?**  For information about this choice, including consent withdrawal, please see our Privacy Policy

Reviewer #1: No

Reviewer #3: **Yes:**  Amer Hamad Issa Abukhalaf

Reviewer #5: **Yes:**  Prof. Irfan Ahmed Rind

**********

[NOTE: If reviewer comments were submitted as an attachment file, they will be attached to this email and accessible via the submission site. Please log into your account, locate the manuscript record, and check for the action link "View Attachments". If this link does not appear, there are no attachment files.]

To ensure your figures meet our technical requirements, please review our figure guidelines: https://journals.plos.org/plosone/s/figures

You may also use PLOS’s free figure tool, NAAS, to help you prepare publication quality figures: https://journals.plos.org/plosone/s/figures#loc-tools-for-figure-preparation.

NAAS will assess whether your figures meet our technical requirements by comparing each figure against our figure specifications.

# Author comment: Author response to Decision Letter 2

**DOI:** 10.1371/journal.pone.0361042.r004

21 Sep 2026

RESPONSE TO THE ACADEMIC EDITOR AND REVIEWERS

Manuscript ID: PONE-D-26-11047R1

Manuscript Title: The Hidden Challenges of Return Migration: Linguistic and Academic Re-Adaptation of Syrian Students to the Arabic Education System

Dear Academic Editor and Reviewers,

We sincerely thank the Academic Editor and the reviewers for their careful second-round evaluation of our manuscript. We are grateful that the substantial improvements made in the previous revision were recognized, and we have addressed each remaining point carefully. In this revision, we refined the methodological description, removed terminology that could imply unsupported conclusions, clarified the sampling and coding chronology, strengthened the contextual and relational interpretation of the findings, added relevant recent literature, and completed a manuscript-wide language and terminology review. All manuscript changes are shown in the tracked-changes version. Below, each reviewer comment is reproduced verbatim, followed by our response and the location of the corresponding revision.

Response to the Academic Editor

Editor comment: Decision: Minor Revision. Both reviewers consider the manuscript interesting, relevant, and methodologically sound, and they recognize its potential contribution to the literature on refugee education and the educational consequences of return migration. Overall, the manuscript has been substantially improved. However, both reviewers have identified several minor issues that should be addressed before the manuscript can be accepted. In particular, the authors are requested to:

Response: We thank the Academic Editor for the encouraging assessment and for identifying the remaining issues so clearly. We addressed each of the five requested areas as detailed below and in our point-by-point responses to Reviewers #1 and #5.

Editor request 1: Provide a clearer justification for the phenomenological design and explain how phenomenological principles informed the data collection and analysis.

Response: After reconsidering the methodological fit in light of the reviewer’s concern, we agreed that retaining a phenomenological label would imply a more specific methodological commitment than was warranted by the written questionnaire and qualitative content-analysis procedures. Rather than adding a retrospective phenomenological justification, we removed the phenomenological designation throughout and now describe the study consistently as an exploratory qualitative study employing qualitative content analysis.

Changes in manuscript: Abstract; Methods; Research Design; Participants; and Limitations and Future Research. The Methods now state, for example, “An exploratory qualitative approach was adopted,” and describe the analytic procedure as qualitative content analysis.

Editor request 2: Revise potentially diagnostic or overly strong psychological terminology and adopt a more contextual and relational interpretation of students’ experiences.

Response: We revised the manuscript throughout to distinguish participants’ reported experiences from psychological interpretation and removed wording that could be read as diagnostic. Theme 4 was reframed around psychosocial and contextual pressures, and we added explicit methodological caution where concentration and emotional difficulties are discussed. We also strengthened the relational/contextual interpretation using Rind (2016) and recent resource-oriented evidence from Opaas et al. (2024).

Changes in manuscript: Abstract; Findings (Theme 4); Discussion; Conclusion; and Recommendations for policy and practice. Theme 4 is now titled “Psychosocial and contextual pressures,” with the categories “emotional strain and disrupted belonging,” “concentration and emotional difficulties in learning,” and “increased family responsibilities.”

Editor request 3: Qualify broad statements about Turkish and Syrian educational systems so that interpretations remain grounded in the participants’ accounts.

Response: We systematically narrowed system-level claims to the evidential level supported by the data. Statements about pedagogy, curriculum, assessment, and classroom practice are now framed as participants’ perceptions and experiences in their previous and current schools rather than as general descriptions of two national education systems.

Changes in manuscript: Abstract; Research Question 3; Findings (Theme 3); Discussion; Conclusion; and Recommendations. Research Question 3 now asks about “the curricular, pedagogical, and assessment practices they experience in their post-return schools.”

Editor request 4: Clarify the procedures and justification concerning data saturation, coding, and the development of the coding framework, and reconcile the relevant tables and supplementary materials.

Response: We clarified the chronology of recruitment, data collection, and coding and removed the earlier prospective-saturation interpretation. The final sample reflected eligible students who were accessible and voluntarily participated during the predefined recruitment period; recruitment was not terminated on the basis of saturation. Coding began only after the data-collection period had ended and the full set of 15 responses was available. We also clarified the analytic hierarchy from meaningful units to initial codes, categories, and four overarching themes. Because saturation was not used prospectively to determine or terminate recruitment, we removed the saturation claim and will not resubmit the previous participant/code and saturation tables as supporting information; retaining a table labelled as saturation could imply a procedure that was not used prospectively. The main manuscript’s substantive thematic tables remain Tables 2–5.

Changes in manuscript: Methods (Participants and Data Analysis); Findings (Tables 2–5). The previous Code_and_Saturation_Tables supporting file is removed from the R2 submission package.

Editor request 5: Carefully proofread the manuscript for grammatical and typographical errors, ensure consistent terminology, and strengthen the discussion by engaging with a few relevant recent studies, including those suggested by the reviewers.

Response: We completed a manuscript-wide proofreading and terminology-consistency review in direct response to this request and Reviewer #1. We standardized the description of the data-collection instrument as an open-ended written questionnaire, corrected residual wording and terminology inconsistencies, and aligned terms such as academic confidence and re-adaptation across sections. We also strengthened the Discussion with two additional recent empirical studies, Ialuna et al. (2024) and Opaas et al. (2024), and incorporated Rind (2016) where it directly supports the requested relational/contextual interpretation.

Changes in manuscript: Throughout the manuscript, especially Methods, Findings, Discussion, and References. Three references were added to the reference list: Rind (2016) [51], Ialuna et al. (2024) [52], and Opaas et al. (2024) [53].

Response to Reviewer #1

Comment: The manuscript addresses an important and underexplored topic concerning the educational reintegration of voluntarily returned Syrian students. The study is well designed, the methodology is appropriate, and the findings provide meaningful theoretical and practical contributions to refugee education and bilingual learning research. The discussion is coherent and the implications are clearly articulated. However, a few minor revisions are recommended before acceptance. The authors should carefully proofread the manuscript for minor grammatical and typographical errors, ensure consistent terminology throughout the text, and strengthen the discussion by explicitly linking the findings to a few additional recent studies. Subject to these minor corrections, the manuscript is suitable for publication.

Response: We sincerely thank the reviewer for the positive assessment and for identifying these final improvements. We conducted a manuscript-wide proofreading and terminology-consistency review rather than limiting the revision to isolated corrections. Among other refinements, we standardized the description of the data-collection instrument as an “open-ended written questionnaire,” corrected an incorrect reference to interviews in the ethics section so that it now states that the students provided verbal assent before completing the questionnaire, used “academic confidence” consistently when referring to confidence rather than self-efficacy, and corrected minor grammatical, typographical, and wording inconsistencies throughout the manuscript. Figure 1 was also revised to ensure full consistency with the terminology used in the Findings. In particular, the figure now uses the revised labels “Difficulties in academic language adaptation,” “Terminological Discontinuity,” “Curriculum Discontinuities,” “Decline in Academic Self-Confidence,” and “Psychosocial and contextual pressures,” together with the revised psychosocial categories “Emotional Strain and Disrupted Belonging” and “Concentration and Emotional Difficulties in Learning.” We also strengthened the Discussion with two additional recent empirical studies: Ialuna et al. (2024), which provides recent evidence on teacher/peer relationships, school belonging, and language-of-schooling challenges among immigrant and refugee children, and Opaas et al. (2024), which supports a resource-oriented interpretation of migrant students’ functioning. Rind (2016) was additionally incorporated in response to Reviewer #5’s request for a relational and contextual perspective.

Changes in manuscript: Manuscript-wide proofreading and terminology review; Fig 1, Discussion paragraphs addressing relational school adjustment and psychosocial/contextual pressures; References [51]–[53].

Response to Reviewer #3

Comment: I have no further Comments. All my comments have been addressed and I am happy with the end results of the paper.

Response: We sincerely thank the reviewer for the encouraging evaluation of the manuscript and for confirming that the previous comments have been fully addressed. We are grateful for the reviewer’s contributions across the review process, which helped improve the clarity and transparency of the manuscript.

Response to Reviewer #5

General comment: This is an interesting paper that shifts attention from refugee integration in host countries to the comparatively neglected educational consequences of return migration, particularly the transition from Turkish-medium to Arabic-medium schooling. The revision has clearly improved the paper, especially in its articulation of the research questions, acknowledgement of limitations, expansion of the theoretical discussion, and description of trustworthiness procedures. However, I still see some issues that should be addressed before publication. I have specifies these issues bellow:

Response: We thank the reviewer for the careful second-round reading and for recognizing the improvements made in the previous revision. We found the remaining methodological and interpretive concerns constructive and have addressed each one below. In particular, we reconsidered the design label rather than defending it retrospectively, narrowed psychological and system-level inferences, clarified the sampling/coding chronology, and strengthened the contextual interpretation of students’ experiences.

Comment 1: The manuscript identifies the study as phenomenological, yet the empirical material was generated through seven open-ended questions administered through Google Forms to 15 students aged 11–14. Although the authors appropriately explain that geographical constraints prevented face-to-face interviews, written responses collected within approximately 15–25 minutes necessarily provide limited opportunities for probing, clarification, and deeper exploration of lived experience. The authors should therefore provide a more convincing explanation of what specifically makes the study phenomenological rather than simply an exploratory qualitative study employing qualitative content analysis. This should include identification of the particular phenomenological orientation adopted and a clearer explanation of how phenomenological principles informed both data collection and interpretation. The authors may find it useful to engage with empirical examples demonstrating how phenomenological inquiry can connect participants’ lived experiences, contextual conditions, and meaning-making processes while maintaining methodological coherence; for example, Soomro et al. (2025) may provide a relevant methodological point of comparison in the field of ESL and education.

Response: We agree with the reviewer’s methodological concern. After reconsidering the relationship among our research questions, written data-collection procedure, and analytic strategy, we concluded that the phenomenological label was more specific than our procedures warranted. We therefore chose not to add a retrospective phenomenological orientation or to defend the label on the basis of participants’ lived experiences alone. Instead, we removed all phenomenological terminology and now describe the study consistently as an exploratory qualitative study using qualitative content analysis. The Research Design section explains that the aim is an in-depth, context-sensitive understanding of students’ experiences and that qualitative content analysis is the analytic procedure used to organize and interpret the written narratives. The Limitation section continues to acknowledge explicitly that written online responses did not permit the depth of follow-up probing available in interviews. We also carefully evaluated Soomro et al. (2025), as suggested. Because the revised manuscript no longer characterizes the design as phenomenological, we did not use this work as methodological support for a design label that we have removed.

Changes in manuscript: Abstract: “Using an exploratory qualitative design…” Methods, Research Design: “An exploratory qualitative approach was adopted.” The subsequent design paragraphs now explain the exploratory, context-sensitive purpose and the role of qualitative content analysis. All phenomenological terminology was removed from the manuscript, including the design and limitation statements.

Comment 2: The manuscript sometimes uses terminology that approaches psychological diagnosis despite the absence of a trauma-specific instrument, clinical assessment, or in-depth psychological interviews. Statements describing concentration difficulties as a “classic effect of trauma,” raised voices as trauma “triggers,” or students as having weakened psychological resilience go beyond what can confidently be inferred from short written accounts. The authors should distinguish more carefully between what participants actually reported and the researchers’ interpretation through the trauma literature. Terms such as “migration-related distress,” “psychosocial strain,” or “experiences potentially consistent with trauma-related responses” would be more defensible. More broadly, the paper would benefit from adopting a relational and contextual interpretation of students’ experiences rather than treating their responses primarily as individual psychological conditions. In this respect, the authors may consider the structure–agency perspective developed by Rind (2016), which emphasises how learners’ experiences emerge through interactions among individual agency, institutional structures, social relationships, and contextual constraints.emerge through interactions among individual agency, institutional structures, social relationships, and contextual constraints. Such a perspective could complement the manuscript’s existing argument that returnees’ difficulties are produced partly through institutional mismatches rather than individual deficits.

Response: We agree and revised this part of the manuscript extensively but selectively, preserving the participant accounts while narrowing the interpretive claims. We removed wording such as “classic effect of trauma,” “triggers,” “deep psychological scars,” and “weakened psychological resilience,” and we no longer infer trauma-specific mechanisms from the written responses. Theme 4 has been reframed as “Psychosocial and contextual pressures.” These terminology changes were also incorporated into Fig. 1 so that the visual representation of the thematic framework fully matches the revised Findings section. The revised Findings explicitly state, for example, that one participant’s account indicates concentration difficulty “but the present data do not establish a trauma-specific mechanism,” and that another participant’s response to raised voices “cannot be interpreted diagnostically” without trauma-specific assessment. We then state explicitly that these accounts are interpreted as psychosocial strain and concentration difficulties rather than as evidence of trauma responses, triggers, or weakened psychological resilience. We also adopted the reviewer’s broader relational/contextual recommendation. Rind (2016) has been incorporated into the Discussion as a conceptual bridge showing how learning experiences can be understood through the interaction of individual agency, classroom community, and institutional influences rather than learner characteristics alone. To strengthen this interpretation with recent empirical evidence, we also added Opaas et al. (2024), whose findings support a resource-oriented perspective that attends to school belonging, resilience, prosocial functioning, and current social conditions alongside stress-related factors.

Changes in manuscript: Abstract; Findings, Fig 1, Theme 4 (“Psychosocial and contextual pressures”); Discussion; Conclusion; Recommendations for policy and practice; References [51] and [53].

Comment 3: Several sections move from students’ individual accounts to rather broad characterisations of Turkish schooling as student-centred and interactive and Syrian schooling as teacher-centred, traditional, transmission-oriented, or authoritarian. For instance, students’ experiences of copying from the board, limited experimentation, and rapid instructional pacing are interpreted as evidence of a broader transition between contrasting pedagogical systems. The data support claims about how these particular students perceived differences between their previous and current classrooms, but a sample of 15 students cannot support general statements about two national education systems. The authors should therefore consistently qualify these interpretations—for example, “participants perceived their current classrooms as more teacher-centred than the classrooms they had previously experienced in Türkiye.” Similar caution is required when discussing curriculum quality, assessment transparency, teacher authority, and pedagogical culture.

Response: We agree with this distinction and have revised the manuscript so that the level of inference matches the level of evidence. We no longer characterize Turkish or Syrian education systems broadly on the basis of the 15 participants’ accounts. Instead, statements about pedagogy, curriculum, assessment, teacher practices, and classroom interaction are explicitly tied to what participants recalled or experienced in their particular previous and current schools. We also revised Research Question 3 so that it no longer presupposes national-system characteristics and instead asks about the curricular, pedagogical, and assessment practices students experience in their post-return schools. For example, the Abstract now refers to “pedagogical practices that participants perceived as more teacher-directed” and “assessment practices that they perceived as unclear.” In the Discussion, the previous cross-system characterization is now framed as a shift “from the more interactive routines participants recalled in their Turkish schools to the more teacher-directed practices they described in their current Syrian classrooms.” Assessment claims are likewise framed as grading criteria and oral assessment practices “that participants perceived as unclear.” These revisions preserve the substantive finding while keeping the interpretation grounded in participants’ accounts.

Changes in manuscript: Abstract; Introduction, Research Question 3; Findings, Theme 3; Discussion; Conclusion; Recommendations for policy and practice.

Comment 4: The response to reviewers states that all twelve sub-codes had emerged by the fifth participant and that no new codes appeared subsequently, which is interpreted as evidence that saturation had been achieved. This claim requires further methodological justification. The authors should explain the order in which responses were collected and analysed, whether coding occurred concurrently with recruitment, how saturation was operationally defined, and whether the sample size was predetermined. In a fixed dataset analysed retrospectively, observing that later participants generated no new codes is not necessarily equivalent to demonstrating prospective theoretical or meaning saturation. Moreover, there appears to be inconsistency between the response letter—which indicates that dedicated participant/code and saturation tables were added—and the clean manuscript, where Tables 2–5 represent the four substantive themes. The authors should reconcile the table numbering and provide the coding/saturation material as supplementary information if necessary. Importantly, all participant-by-code entries must derive directly from the underlying data rather than being retrospectively completed to create a more symmetrical coding matrix.

Rind, I. A. (2016). Conceptualizing students’ learning experiences in English as a second language in higher education from structure and agency. Cogent Social Sciences, 2, 1191978.

Soomro, R. B. K., Khan, R. A., & Rind, I. A. (2025). Exploring ESL teachers’ experiences about ICT-based 4Cs skills: A phenomenological study. Discover Education, 4(12).

Response: We thank the reviewer for drawing this important distinction between a retrospective pattern in a fixed dataset and prospective saturation used to determine recruitment. The coding-progression and saturation material was added in the previous revision in direct response to a reviewer request for greater transparency regarding how the coding developed across participants. The present comment made clear, however, that describing the later absence of new codes as “data saturation” could imply that saturation was used prospectively to determine sample adequacy or to stop recruitment. That was not the procedure used in this study. We have therefore revised the methodological reporting rather than attempting to strengthen a saturation claim that does not describe the recruitment process. The Participants section now clarifies that the final sample reflected eligible students who were accessible and voluntarily participated during the predefined recruitment period and that recruitment was not terminated on the basis of prospective saturation. The Data Analysis section now states that coding commenced only after the data-collection period had ended and the complete set of 15 responses was available; coding therefore did not occur concurrently with participant recruitment. All 15 responses were analysed in a fixed analytic sequence, and that sequence played no role in determining recruitment or sample size. We have removed the statement that saturation was reached by the fifth participant. We also reconciled the table issue. The main manuscript’s Tables 2–5 remain the four substantive thematic tables. Because prospective saturation was not used to determine recruitment, we do not resubmit the previous participant/code and saturation tables as supplementary information. This avoids retaining a “saturation” representation that could imply a prospective sampling procedure that was not used. Transparency regarding the coding framework is instead provided directly in the revised Data Analysis section, which now describes the progression from meaningful units to initial codes, categories, and four overarching themes, together with the thematic tables and representative participant extracts in the Findings. This revision changes the methodological reporting only; it does not alter the dataset, coding, themes, or substantive findings.

Changes in manuscript: Methods, Participants and Data Analysis. The prospective saturation claim was removed. The previous Code_and_Saturation_Tables supporting file will not be resubmitted. Main-text Tables 2–5 remain the four substantive theme tables.

Reference-list changes in this revision

As requested in the decision letter, we note the following additions to the reference list:

[51] Rind IA. Conceptualizing students’ learning experiences in English as second language in higher education from structure and agency. Cogent Soc Sci. 2016;2(1):1191978. doi: 10.1080/23311886.2016.1191978.

[52] Ialuna F, Civitillo S, McElvany N, Leyendecker B, Jugert P. Resilience in multicultural classrooms: School relationships can protect the school adjustment of immigrant, refugee and non-immigrant children. Br J Educ Psychol. 2024;94(4):1271-93. doi: 10.1111/bjep.12713.

[53] Opaas M, Wentzel-Larsen T, Nissen A, Hilden PK, Punamäki RL, Spaas C, et al. Migrant students’ self-reported executive function skills in relation to mental health, postmigration stress, and positive appraisals of self and the social context. Eur J Psychotraumatol. 2024;15(1):2430923. doi: 10.1080/20008066.2024.2430923.

Regarding the reviewer-suggested Soomro et al. (2025) reference: we evaluated the study as suggested. Because the revised manuscript no longer identifies the design as phenomenological, we did not cite it as methodological support for a phenomenological orientation that has been removed.

We thank the Academic Editor and the reviewers once again for their careful and constructive feedback. We believe that the revisions have resolved the remaining methodological and interpretive concerns while preserving the study’s evidence-based contribution. We hope that the revised manuscript is now suitable for publication in PLOS ONE.

Sincerely,

The Authors

Attachment Submitted filename: Response letter_20Sep.docx

# Editor report: Decision Letter 2

**DOI:** 10.1371/journal.pone.0361042.r005

**Authors:** Ömer Gökhan Ulum

30 Sep 2026

Dear Dr. Dolapcioglu,

Thank you for submitting your manuscript to PLOS One. After careful consideration, we feel that it has merit but does not fully meet PLOS One’s publication criteria as it currently stands. Therefore, we invite you to submit a revised version of the manuscript that addresses the points raised during the review process.

==============================

Your article makes valuable contributions by addressing a neglected yet extremely important topic, such as the educational integration of Syrian students who have voluntarily returned; however, the methodological and conceptual shortcomings noted in the peer review reports must be addressed prior to publication. In this regard, the authors should: 1) more convincingly justify the study’s phenomenological orientation by taking into account the limitations of the data collection process, which was conducted via short written responses on Google Forms; 2) replace psychological/trauma-focused terminology used without clinical evaluation with more cautious and context-specific concepts (including the structure-agency perspective); 3) avoid making bold claims about the general education systems of the two countries based on a limited sample of 15 participants and instead phrase their statements in a more cautious manner, and 4) transparently clarify the methodological rationale for data saturation and resolve inconsistencies in the tables. Please make the necessary revisions in light of the reviewers' feedback and upload your article to the system before the deadline.

==============================

Please submit your revised manuscript by Nov 14 2026 11:59PM. If you will need more time than this to complete your revisions, please reply to this message or contact the journal office at plosone@plos.org. When you're ready to submit your revision, log on to https://www.editorialmanager.com/pone/ and select the 'Submissions Needing Revision' folder to locate your manuscript file.

- A letter that responds to each point raised by the academic editor and reviewer(s). You should upload this letter as a separate file labeled 'Response to Reviewers'.
- A marked-up copy of your manuscript that highlights changes made to the original version. You should upload this as a separate file labeled 'Revised Manuscript with Track Changes'.
- An unmarked version of your revised paper without tracked changes. You should upload this as a separate file labeled 'Manuscript'.

If you would like to make changes to your financial disclosure, please include your updated statement in your cover letter. Guidelines for resubmitting your figure files are available below the reviewer comments at the end of this letter.

If applicable, we recommend that you deposit your laboratory protocols in protocols.io to enhance the reproducibility of your results. Protocols.io assigns your protocol its own identifier (DOI) so that it can be cited independently in the future. For instructions see: https://journals.plos.org/plosone/s/submission-guidelines#loc-laboratory-protocols. Additionally, PLOS One offers an option for publishing peer-reviewed Lab Protocol articles, which describe protocols hosted on protocols.io. Read more information on sharing protocols at https://plos.org/protocols?utm_medium=editorial-email&utm_source=authorletters&utm_campaign=protocols.

As the corresponding author, your ORCID iD is verified in the submission system and will appear in the published article. PLOS supports the use of ORCID, and we encourage all coauthors to register for an ORCID iD and use it as well. Please encourage your coauthors to verify their ORCID iD within the submission system before final acceptance, as unverified ORCID iDs will not appear in the published article. *Only*  the individual author can complete the verification step; PLOS staff *cannot*  verify ORCID iDs on behalf of authors.

We look forward to receiving your revised manuscript.

Kind regards,

Ömer Gökhan Ulum

Academic Editor

PLOS One

Journal Requirements:

If the reviewer comments include a recommendation to cite specific previously published works, please review and evaluate these publications to determine whether they are relevant and should be cited. There is no requirement to cite these works unless the editor has indicated otherwise.

Please review your reference list to ensure that it is complete and correct. If you have cited papers that have been retracted, please include the rationale for doing so in the manuscript text, or remove these references and replace them with relevant current references. Any changes to the reference list should be mentioned in the rebuttal letter that accompanies your revised manuscript. If you need to cite a retracted article, indicate the article’s retracted status in the References list and also include a citation and full reference for the retraction notice.

Additional Editor Comments

Your article makes valuable contributions by addressing a neglected yet extremely important topic, such as the educational integration of Syrian students who have voluntarily returned; however, the methodological and conceptual shortcomings noted in the peer review reports must be addressed prior to publication. In this regard, the authors should: 1) more convincingly justify the study’s phenomenological orientation by taking into account the limitations of the data collection process, which was conducted via short written responses on Google Forms; 2) replace psychological/trauma-focused terminology used without clinical evaluation with more cautious and context-specific concepts (including the structure-agency perspective); 3) avoid making bold claims about the general education systems of the two countries based on a limited sample of 15 participants and instead phrase their statements in a more cautious manner, and 4) transparently clarify the methodological rationale for data saturation and resolve inconsistencies in the tables. Please make the necessary revisions in light of the reviewers' feedback and upload your article to the system before the deadline.

[Note: HTML markup is below. Please do not edit.]

[NOTE: If reviewer comments were submitted as an attachment file, they will be attached to this email and accessible via the submission site. Please log into your account, locate the manuscript record, and check for the action link "View Attachments". If this link does not appear, there are no attachment files.]

To ensure your figures meet our technical requirements, please review our figure guidelines: https://journals.plos.org/plosone/s/figures

You may also use PLOS’s free figure tool, NAAS, to help you prepare publication quality figures: https://journals.plos.org/plosone/s/figures#loc-tools-for-figure-preparation.

NAAS will assess whether your figures meet our technical requirements by comparing each figure against our figure specifications.

# Author comment: Author response to Decision Letter 3

**DOI:** 10.1371/journal.pone.0361042.r006

2 Oct 2026

We express our sincere gratitude to the Academic Editor and the reviewers for their meticulous evaluation. We appreciate their recognition of the progress made in the previous revision and have diligently addressed all remaining feedback. Specifically, this version features refined methodological details, the removal of over-extended claims, clarified sampling and coding timelines, deepened contextual interpretations, updated references, and a comprehensive language polish. All modifications are highlighted in the tracked-changes document. Each reviewer comment is addressed individually, detailing our response and the exact location of the revision.

Attachment Submitted filename: Response_letter_20Sep_auresp_3.docx

# Editor report: Decision Letter 3

**DOI:** 10.1371/journal.pone.0361042.r007

**Authors:** Ömer Gökhan Ulum

6 Oct 2026

The Hidden Challenges of Return Migration: Linguistic and Academic Re-Adaptation of Syrian Students to the Arabic Education System

PONE-D-26-11047R3

Dear Author(s),

We’re pleased to inform you that your manuscript has been judged scientifically suitable for publication and will be formally accepted for publication once it meets all outstanding technical requirements.

Within one week, you’ll receive an e-mail detailing the required amendments. When these have been addressed, you’ll receive a formal acceptance letter and your manuscript will be scheduled for publication.

An invoice will be generated when your article is formally accepted. Please note, if your institution has a publishing partnership with PLOS and your article meets the relevant criteria, all or part of your publication costs will be covered. Please make sure your user information is up-to-date by logging into Editorial Manager at Editorial Manager® and clicking the ‘Update My Information' link at the top of the page. For questions related to billing, please contact billing support.

If your institution or institutions have a press office, please notify them about your upcoming paper to help maximize its impact. If they’ll be preparing press materials, please inform our press team as soon as possible -- no later than 48 hours after receiving the formal acceptance. Your manuscript will remain under strict press embargo until 2 pm Eastern Time on the date of publication. For more information, please contact onepress@plos.org.

Kind regards,

Ömer Gökhan Ulum

Academic Editor

PLOS One

Additional Editor Comments (optional):

While this article focuses on a highly topical and underexplored area—such as the educational integration of students in the voluntary return process—it earned publication thanks to the exceptional academic integrity and meticulousness the authors demonstrated throughout the revision process. The authors have completely eliminated the methodological oversimplifications present in the first version (such as the claim of a phenomenological approach based on written forms and the assumptions of retrospective saturation), limited national-level generalizations to participants’ perceptions, and, by filtering out clinical and diagnostic discourses, grounded the study in a robust structure-agency and social-relational framework. Supported by current and high-quality literature, and with methodological transparency and conceptual depth maximized, this study meets our journal’s scientific standards and makes a lasting contribution to the field.

Reviewers' comments:

# Editor report: Acceptance letter

**DOI:** 10.1371/journal.pone.0361042.r008

**Authors:** Ömer Gökhan Ulum

PONE-D-26-11047R3

PLOS One

Dear Dr. Dolapcioglu,

I'm pleased to inform you that your manuscript has been deemed suitable for publication in PLOS One. Congratulations! Your manuscript is now being handed over to our production team.

At this stage, our production department will prepare your paper for publication. This includes ensuring the following:

* All references, tables, and figures are properly cited

* All relevant supporting information is included in the manuscript submission,

* There are no issues that prevent the paper from being properly typeset

You will receive further instructions from the production team, including instructions on how to review your proof when it is ready. Please keep in mind that we are working through a large volume of accepted articles, so please give us a few days to review your paper and let you know the next and final steps.

Lastly, if your institution or institutions have a press office, please let them know about your upcoming paper now to help maximize its impact. If they'll be preparing press materials, please inform our press team within the next 48 hours. Your manuscript will remain under strict press embargo until 2 pm Eastern Time on the date of publication. For more information, please contact onepress@plos.org.

You will receive an invoice from PLOS for your publication fee after your manuscript has reached the completed accept phase. If you receive an email requesting payment before acceptance or for any other service, this may be a phishing scheme. Learn how to identify phishing emails and protect your accounts at https://explore.plos.org/phishing.

If we can help with anything else, please email us at customercare@plos.org.

Thank you for submitting your work to PLOS One and supporting open access.

Kind regards,

PLOS One Editorial Office Staff

on behalf of

Dr. Ömer Gökhan Ulum

Academic Editor

PLOS One
